# Controlled aggregation of Pt/PtH/Rh/RhH doped silver superatomic nanoclusters into 16-electron supermolecules†

**DOI:** 10.1039/d4sc02920h

**Published:** 2024-08-22

**Authors:** Tzu-Hao Chiu, Michael N. Pillay, Ying-Yann Wu, Yoshiki Niihori, Yuichi Negishi, Jie-Ying Chen, Yuan Jang Chen, Samia Kahlal, Jean-Yves Saillard, C. W. Liu

**Affiliations:** a Department of Chemistry, National Dong Hwa University Hualien 97401 Taiwan Republic of China chenwei@gms.ndhu.edu.tw; b Department of Applied Chemistry, Tokyo University of Science 1-3 Kagurazaka, Shinjuku Tokyo 162-8601 Japan; c Department of Chemistry, Fu Jen Catholic University New Taipei City 24205 Taiwan Republic of China; d Univ Rennes, CNRS, ISCR-UMR 6226 F-35000 Rennes France

## Abstract

The assembly of discrete superatomic nanoclusters into larger constructs is a significant stride towards developing a new set of artificial/pseudo-elements. Herein, we describe a novel series of 16-electron supermolecules derived from the combination of discrete 8-electron superatomic synthons containing interstitial hydrides as vertex-sharing building blocks. The symmetric (RhH)_2_Ag_33_[S_2_P(OPr)_2_]_17_ (1) and asymmetric PtHPtAg_32_[S_2_P(OPr)_2_]_17_ (2) are characterized by ESI-MS, SCXRD, NMR, UV-vis absorption spectra, electrochemical and computational methods. Cluster 1 represents the first group 9-doped 16-electron supermolecule, composed of two icosahedral (RhH)@Ag_12_ 8-electron superatoms sharing a silver vertex. Cluster 2 results from the assembly of two distinct icosahedral units, Pt@Ag_12_, and (PtH)@Ag_12_. In both cases, the presence of the interstitial hydrides is unprecedented. The stability of the supermolecules is investigated, and 2 spontaneously transforms into Pt_2_Ag_33_[S_2_P(OPr)_2_]_17_ (3) with thermal treatment. The lability of the hydride within the icosahedral framework in solution at low-temperature was confirmed by the VT-NMR.

## Abstract 2

The controlled assembly of superatoms into well-defined supermolecules represents the next evolutionary step in the field of atomically precise nanoclusters.

## Graphical abstract

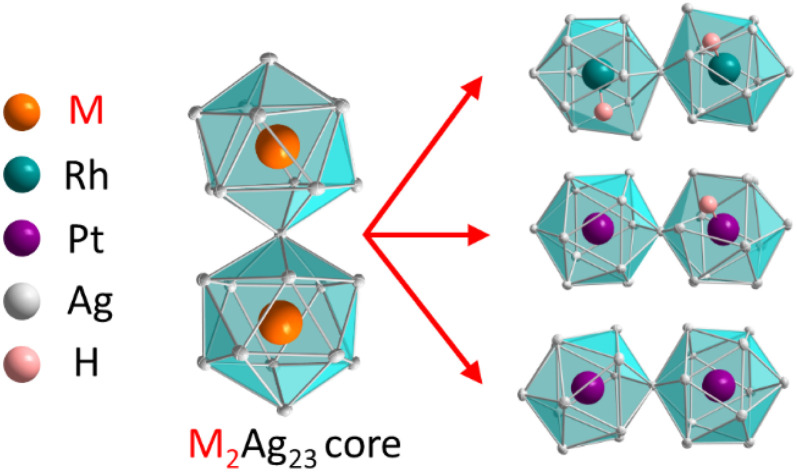

## Introduction

**Fig. 1:**
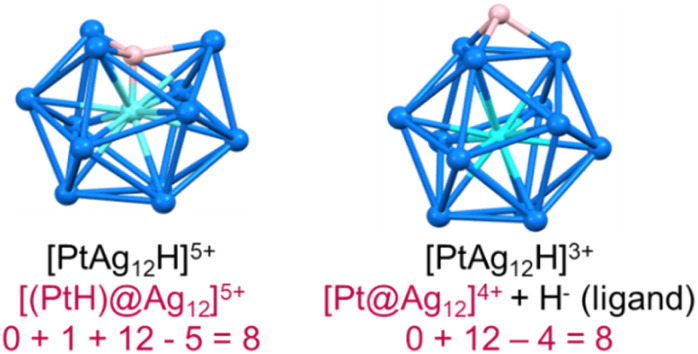
The 8-electron superatomic systems [PtHAg_12_]^5+^ (left) and [PtAg_12_H]^3+^ (right).

In recent years, superatomic nanoclusters (NCs) containing hydrides encapsulated within their inner core have gained significant attention due to their highly unique bonding and promising properties.^1–11^ Isolating and identifying such clusters poses a considerable challenge. Nevertheless, studying these molecules is crucial for understanding their growth, synthesis, and stability. As more hydride-containing nanoclusters are discovered, the importance of the hydride position becomes increasingly evident, as hydrides in encapsulated locations exhibit properties different from traditional hydride ligands lying at the NC surface. Let us, for example, consider the centered icosahedral 8-electron [(PtH)@Ag_12_]^5+^ core of the stable PtHAg_19_(dtp)_12_ (dtp = S_2_P(O^*n*^Pr)_2_) NC.^4^ The formal addition of two extra electrons to it results in the expulsion of the hydride outside the cage, forming an 8-electron [Pt@Ag_12_]^4+^ coordinated by a regular outer hydride (H^−^) ligand, *i.e.*, {[Pt@Ag_12_](μ_3_-H)}^3+^ (Fig. 1).

In the same way, as atoms combine to build molecules, superatomic clusters can assemble to form superatomic molecules (supermolecules). Such assemblies, described as “clusters of clusters” by Teo *et al.* in the last century, continue to attract attention.^12^ Most are one-dimensional assemblies,^13,14^ although two-dimensional^15^ and even three-dimensional examples have been reported.^16,17^ The stability of such supermolecules is governed by electron counting rules that are similar to those of simple molecules (octet rule, for example).^18–21^ Supermolecules of which the inner core is composed of two centered icosahedra sharing a vertex, an edge, or a face, are well-known.^13,22–45^ Most are composed of two quasi-independent closed-shell 8-electron superatoms, making them equivalent to the van der Waals dimers of Ne (16 valence electrons). Surface engineering,^33,34^ and heterometal doping^35^ have been common strategies to tune the properties of these 16-electron supermolecules. To our knowledge, no supermolecule containing one or several encapsulated hydrides has been reported so far. Below are the first examples of these unique supermolecules that contain interstitial hydrides, resulting in electronic stability. RhH-doped superatoms are extremely rare in current literature, and the 16-electron silver-rich homoleptic NCs of the type (RhH)_2_Ag_33_[S_2_P(O^*n*^Pr)_2_]_17_(1) is the first of its kind. Its core comprises two vertex-sharing (RhH)@Ag_12_ centered icosahedral motifs.

The platinum-doped system of the type PtHPtAg_32_[S_2_P(O^*n*^Pr)_2_]_17_ (2) is composed of two different 8-electron units: a (PtH)@Ag_12_ and a Pt@Ag_12_. ESI-MS and SCXRD identified the NC compositions, and the presence of hydrides was confirmed by ^1^H NMR spectroscopy. Furthermore, their electronic structure was analyzed by DFT. This is the first example of hydrides being incorporated into 16-electron bi-icosahedral cores and the first group 9 metal-doped 16-electron supermolecule. The low-temperature emission approaches the sort after near-infrared region, which is currently under intense investigation.^36^ This provides new avenues for future research on these supermolecules.

## Results and discussion

**Fig. 2:**
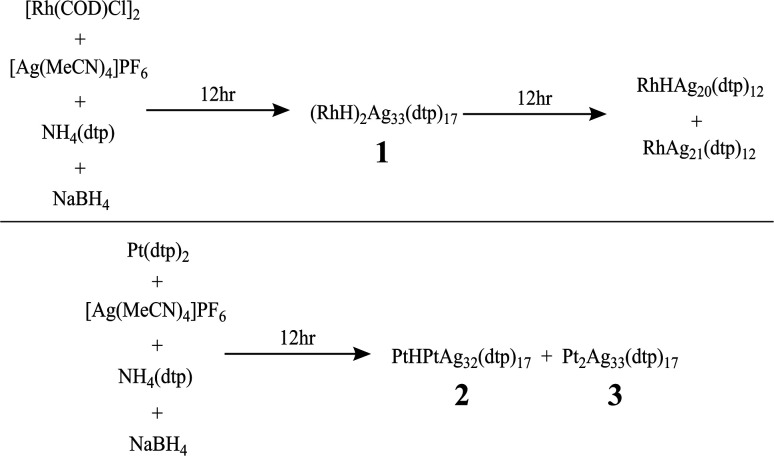
Synthesis and decomposition of the hydride-containing supermolecules.

The synthesis of hydride-doped NCs depends on several key factors, the reaction time of which is crucial. The sequence of the reactions is illustrated in Fig. 2, with the isolated NC highly dependent on the duration of the reaction. Compound 1 was synthesized using a one-pot co-reduction method involving the reaction of [Ag(MeCN)_4_]PF_6_, NH_4_[S_2_P(OPr)_2_], [Rh(COD)Cl]_2_, and NaBH_4_. The purified product was crystallized by slow evaporation in methanol, resulting in black block-shaped crystals after one week. A similar procedure was adopted for synthesizing 2, with Pt[S_2_P(OPr)_2_]_2_ as the dopant source. The kernel transformation of 2 into 3 was experimentally tracked by ^31^P NMR (Fig. S1†). In contrast, heating 1 at 333 K for 120 min does not result in the retention of the bi-icosahedra and results in the isolation of two independent 8-electron NCs of the type [RhHAg_20_(dtp)_12_] and [RhAg_21_(dtp)_12_] (Fig. S2†).

**Fig. 3:**
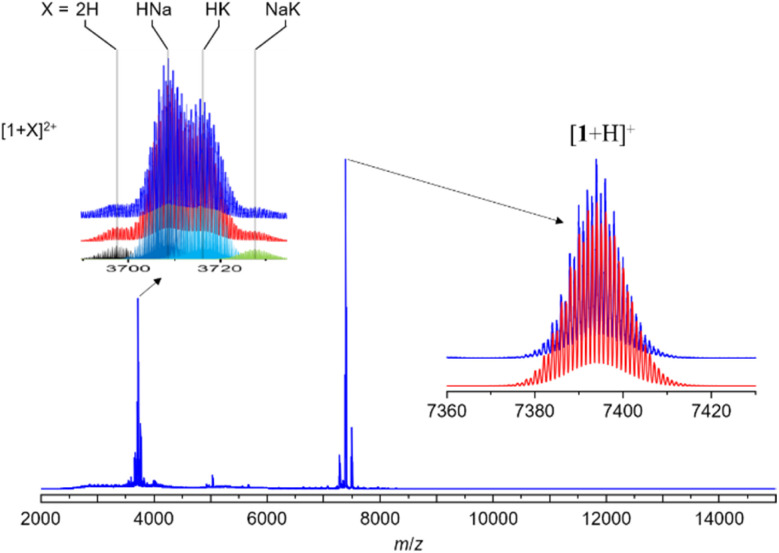
Positive-mode ESI mass spectrum of 1. Insets: experimental (top) and simulated (down).

The assigned compositions of 1–3 were confirmed by ESI-MS spectroscopy. In the ESI-MS spectrum of 1, distinct signals were observed around 7500 Da and 3700 Da, with the strongest at 7392.983 Da (calc. 7392.990) corresponding to [1 + H]^+^. Additionally, signals for dicationic adducts, [1 + 2H]^2+^, [1 + H + Na]^2+^, [1 + H + K]^2+^, and [1 + Na + K]^2+^ were observed. ([1 + 2H]^2+^: exp. 3697.013 calc. 3696.998; [1 + H + Na]^2+^: exp. 3708.051 calc. 3707.989; [1 + H + K]^2+^: exp. 3715.022 calc. 3715.976; [1 + Na + K]^2+^: exp. 3726.485 calc. 3726.967) (Fig. 3). In the ESI-MS spectrum of 2, the signal at 3841.954 Da corresponds to [2 + 2Ag]^2+^ (calc. 3841.987), and the signal at 3895.446 Da corresponds to [3 + 2Ag]^2+^ (Fig. S3†).

**Fig. 4:**
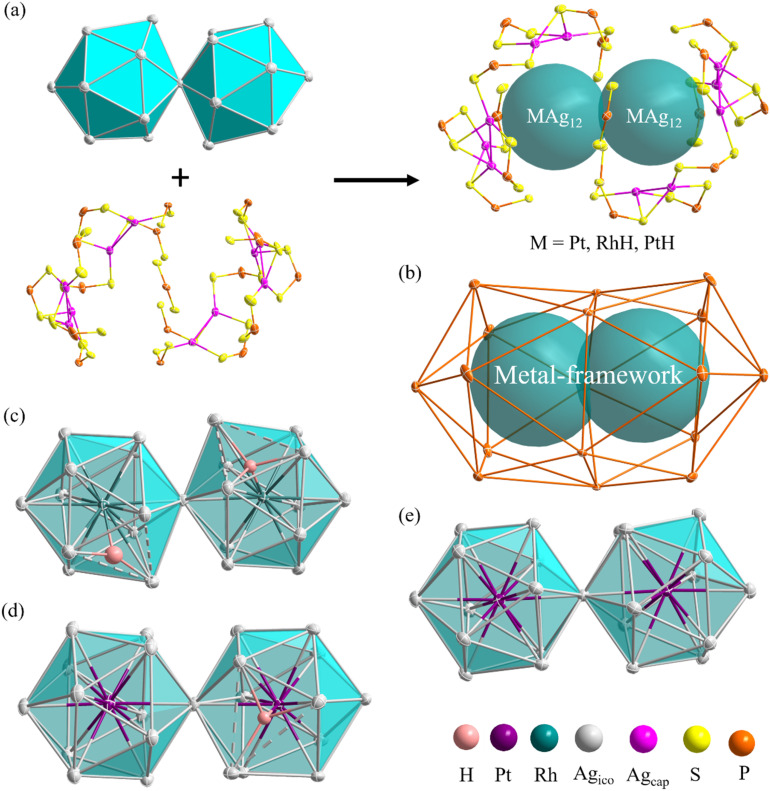
(a) The disassembly diagram of a superatomic molecule and passivating layer. (b) Interpenetrated icosahedra composed of 17 P atoms (c) the (RhH)_2_Ag_23_ bi-icosahedral core of 1 (d) the (PtH)PtAg_23_ bi-icosahedral core of 2 (e) the Pt_2_Ag_23_ bi-icosahedral core of 3.

The molecular structures of compounds 1–3 were determined by SCXRD analysis (see ESI†). Selected metrical data are provided in Table 1. The structure of 1 (Fig. 4) can be viewed as an (RhH)_2_Ag_23_ core, made of two vertex-sharing (RhH)@Ag_12_ centered icosahedra and passivated by 10 capping silver atoms (Ag_cap_) and 17 dithiophosphate ligands (dtp) (Fig. 4a). These ligands are organized in a 1 : 5 : 5 : 5 : 1 ratio to form a large interpenetrated P_17_ icosahedron (Fig. 4b). The ten outer Ag_cap_ atoms are distributed in a 3 : 2 : 2 : 3 sequence, capping three among the five triangular faces at each end of the icosahedra and two intermediate triangular faces (Fig. S4†). The two (RhH)@Ag_12_ centered icosahedra composing the NC core are distorted, with one enlarged Ag_3_ triangular face (Ag⋯Ag > 2.9 Å). The continuous symmetry measure (CSM)^34^ values of 0.68 and 0.55 can evaluate the icosahedral distortion, which indicates substantial deviation from ideal *I*_h_ symmetry.

**Table 1:** Selected experimental bond distances (Å) and angles (deg) for compounds 1–3

NC	1		2		3	
Superatomic core	RhH@Ag_12_	RhH@Ag_12_	Pt@Ag_12_	PtH@Ag_12_	Pt@Ag_12_	Pt@Ag_12_
CSM	0.68	0.55	0.06	0.73	0.1	0.1
M_cent_–Ag_ico_(av.)	2.691(1)–3.117(2), [2.792(6)]	2.688(1)–3.082(2), [2.790(6)]	2.712(1)–2.790(1), [2.756(4)]	2.7065(9)–3.077(1), [2.808(3)]	2.714(1)–2.842(1), [2.759(2)]	2.714(1)–2.842(1), [2.759(2)]
Ag_ico_–Ag_ico_(av.)	2.718(1)–3.760(2), [2.941(9)]	2.743(2)–3.726(2), [2.937(9)]	2.824(1)–3.022(1), [2.898(7)]	2.759(1)–3.782(1), [2.956(7)]	2.805(1)–3.020(1), [2.895(4)]	2.805(1)–3.020(1), [2.895(4)]
Ag_ico_–Ag_cap_(av.)	2.829(2)–3.158(2), [2.965(7)]	2.861(2)–3.122(2), [2.963(6)]	2.843(2)–3.184(2), [2.972(6)]	2.884(2)–3.111(2), [2.004(5)]	2.847(1)–3.228(1), [2.998(4)]	2.847(1)–3.228(1), [2.998(4)]
M_cent_–H	1.9(2)	1.6(1)	—	1.68(2)	—	—
Ag–H(av.)	1.9(2)–1.9(2), [1.9(3)]	1.8(2)−2.3(1), [2.0(3)]	—	2.06(2)−2.08(2), [2.07(3)]	—	—
Torsion angle between the superatoms	22		16		19	
Bond angles between the superatoms	177		175		175	

The icosahedra are arranged in a partial gauche rotational conformation with an average torsion angle of 22 degrees. Their hydride positions were identified through residual electron density maps and refined without constraints. They correspond to the tetrahedral cavities defined by the central Rh and the enlarged Ag_3_ faces. Inside these cavities, the two hydrides are found to have different coordination modes, *i.e.*, a μ_4_ tetrahedral (RhAg_3_) and a μ_3_ pyramidal (RhAg_2_) connectives (Table 1). It is important to remember that the SCXRD location of such hydrides should be considered approximate. Not considering the somewhat different coordination modes of the hydrides, the overall ideal symmetry of 1 is *C*_2_, with the *C*_2_ axis perpendicular to the Rh⋯Rh vector containing one P atom and the Ag vertex shared by the two icosahedra (Fig. S5†).

Compound 2 exhibits an unsymmetrical bi-icosahedral core made of two different vertex-sharing centered icosahedra, namely, (PtH)@Ag_12_ and Pt@Ag_12_ (Fig. 4d). The hydride was identified through residual electron density maps and refined with partial constraint. It occupies an Ag_3_Pt tetrahedral cavity (μ_4_ coordination mode) in one of the icosahedra, causing, as in 1, the enlargement of the corresponding Ag_3_ face. This distortion is responsible for the relatively large associated CSM value of 0.73 (same order of magnitude as in 1), whereas that of the other icosahedron (0.06) denotes a regular structure. As in 1, the icosahedra are arranged in a gauche conformation, with an average torsion angle of 16 degrees. Overall, the passivating outer shell of 2 resembles that of 1, except that there are only 9 Ag_cap_ atoms in 2, organized in a 3 : 2 : 2 : 2 sequence, the missing Ag_cap_ being associated with the (PtH)@Ag_12_ icosahedron (Fig. S4†).

The overall structure of 3 is similar to that of 1, and its ideal symmetry is also *C*_2_. Yet, this time, the Pt_2_Ag_23_ core (Fig. 4e) is composed of two regular Pt@Ag_12_ icosahedra (CSM = 0.10 for both icosahedra). The molecular structure of 3 has been reported;^29^ however, the change in the synthetic route leads to a comparatively different packing arrangement in the crystal lattice. This atypical case of polymorphism is not driven by the inclusion of solvates or a change in counterion (Fig. S6†). The crystal packing in the presently reported polymorph is composed of two different layers, A and B, A being more densely packed than B. In contrast, the previously reported polymorph of 3 is only composed of type B layers (Fig. S6†).

**Fig. 5:**
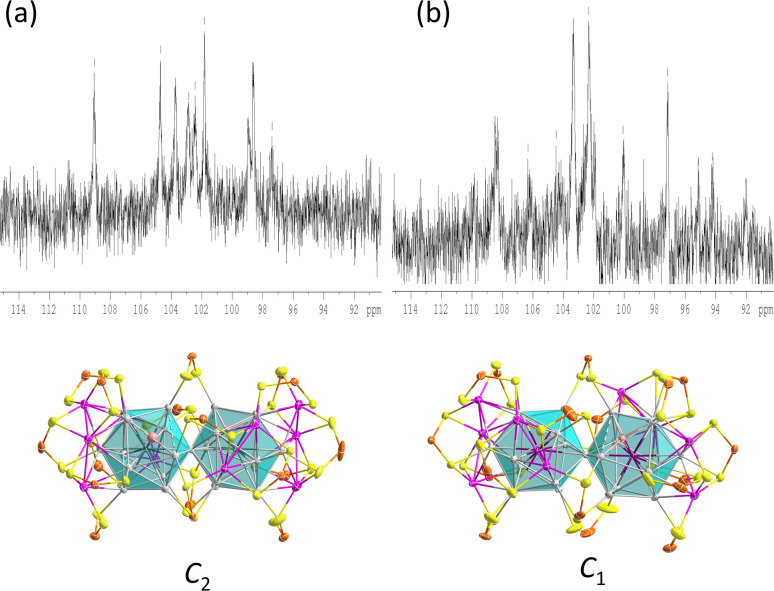
The ^31^P NMR spectrum of 1 (a) and 2 (b) at 173 K, and the corresponding symmetry.

In the similar *C*_2_ structures of 1 and 3, the 17 ligands experience nine distinct chemical environments. Consistently, the ^31^P NMR spectrum of compound 1 (Fig. 5 and S7†) is similar to that of compound^3,29^ with significantly broad resonances observed at 104.7 ppm and 96.8 ppm, along with a sharp peak at 101.0 ppm (Fig. S7†).

In the variable temperature (VT) ^31^P NMR spectra of 1, when the temperature is lowered to 213 K, the three peaks split into eight peaks of equal intensity and one with half intensity, corresponding to the ligand environments observed in the solid-state structure. The ^1^H NMR hydride signals are found at −12.1 ppm, with a hydride-to-ligand integration ratio of 2 : 17 (Fig. S8†). The hydride resonance of 1 remains a broad peak even at low temperatures, indicating substantial hydride mobility within the metallic framework.

The ^31^P NMR spectrum of compound 2, owing to its asymmetric structure, exhibits three sets of broad peaks at 106.0 ppm, 104.7 ppm, and 96.2 ppm, accompanied by a sharp peak at 101.0 ppm (Fig. S9†). The VT ^31^P NMR spectrum at 173 K shows a splitting into eleven peaks of varying intensity (Fig. 5 and S13†). Because of peak overlapping, it is not possible to discern individual resonances for each of the 17 symmetrically inequivalent nuclei. The hydride resonance is observed around −10.4 ppm, flanked by Pt satellites with a coupling constant of 512 Hz, confirming the interaction of the hydride with the central Pt atom (Fig. S14†). In the VT ^1^H NMR spectra of 2, the hydride resonance does not split due to coupling to the silver nuclei as observed in PtHAg_19_(dtp)_12_,^4^ but remains a broad peak with Pt satellites at 173 K (Fig. S16†). This absence of splitting is likely related to the rapid exchange of hydride positions within the metal core cavity. The ^195^Pt{^1^H} NMR spectrum of 2 exhibits a broad singlet at −7712 ppm at 173 K, which represents an upfield shift compared to most examples, except for PtHAg_19_(dtp)_12_.^4^ (Fig. S14–S16†).

Hydride-containing nanoclusters usually exhibit poor thermal stability, as the hydrides tend to dissociate from the clusters and reduce the metal upon further heating (Fig. 2). The thermal stability of compound 1 is better than that of compound 2. Compound 1 is persistent in THF at 60 °C for at least three hours. After six hours, it completely decomposes into RhAg_21_(dtp)_12_ and RhHAg_20_(dtp)_12_ (Fig. S2†). After heating, compound 2 decomposes to yield compound 3 and PtAg_20_(dtp)_12_ after one hour. After two hours of heating, it completely decomposes, leaving only 3 and PtAg_20_(dtp)_12_. (Fig. S1†).

**Fig. 6:**
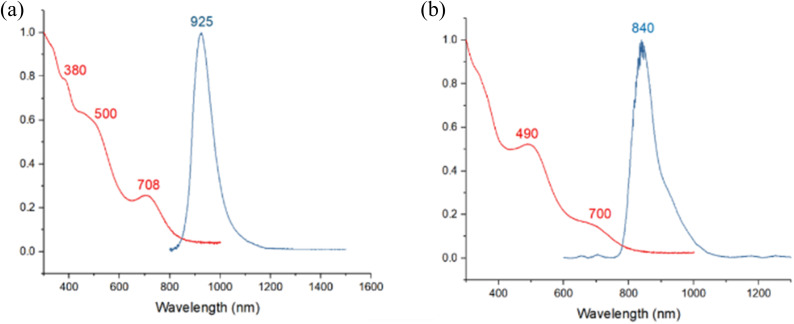
Absorption (2-MeTHF, RT, red) and emission (2-MeTHF glass, 77 K, blue) spectra of (a) 1 (b) 2.

The absorption spectrum of 1 displays three bands at 380 nm, 500 nm, and 708 nm, similar to 3. At 77 K, the photoluminescence spectrum shows a prominent signal near 925 nm in the near-infrared region, exhibiting a noticeable redshift compared to 3. The absorption spectrum of 2 shows a slight blueshift compared to 3, with two absorption bands at 490 nm and 700 nm. The photoluminescence spectrum at 77 K displays a slight redshift at 840 nm compared to 3 (Fig. 6). The photoluminescence decay of samples 1–3 exhibits a single exponential decay pattern, indicating a singular lifetime of 50 μs (1), 25 μs (2), and 21 μs (3) (Fig. S17–S19†), suggesting phosphorescence. The photoluminescence quantum yields (PLQY) of compounds 1, 2, and 3 are 5.5, 22, and 26%, respectively. This indicates that the Pt atom favors radiative decay, achieving superior PLQY even with the same ligand shell.

**Fig. 7:**
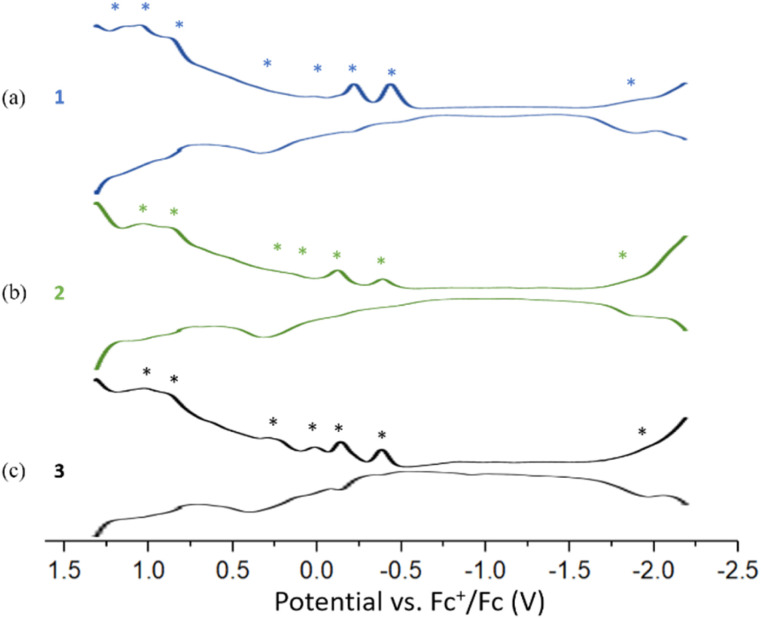
DPV curves of (a) 1 (b) 2 (c) 3 were obtained in a 0.1 M DCM solution of Bu_4_NPF_6_ at 233 K. (Potential increments: 2 mV; pulse amplitude: 50 mV; pulse width: 50 ms; sampling width: 25 ms; pulse period: 100 ms.).

The Differential Pulse Voltammetry (DPV) of 1, 2, and 3 were performed in DCM at 233 K. Compound 1 displays seven oxidation peaks at −0.44(O1), −0.22(O2), 0.15(O3), 0.33(O4), 0.88(O5), 1.04(O6) and 1.15(O7) and one re-duction peak at −1.90(R1). Compound 2 shows six oxidation peaks at −0.38(O1), −0.12(O2), 0.15(O3), 0.33(O4), 0.87(O5) and 1.03(O6) and one reduction peak at −1.88(R1). Compound 3 displays four oxidation peaks at −0.38(O1), −0.14(O2), 0.01(O3), 0.24(O4), 0.89(O5) and 1.03(O6) and one reduction peak at −1.96(R1) (Fig. 7). The three DPV voltammograms are similar, and the corresponding electrochemical gaps are significantly smaller than for related 8-electron superatoms.^7,29^ (Fig. S20–S21†).

Compounds 1 and 2 were investigated by DFT calculations at the BP86/Def2-TZVP level of calculations (see Computational details in the ESI†). For the sake of computational limitations, the dtp ligands were replaced by S_2_PH_2_ models, a simplification which has been proved to be reasonable in many past investigations.^3,7,8,29,46–54^ Their geometries were fully optimized without any symmetry constraint. The resulting structures are in good agreement with their SCXRD counter-parts. Selected computed data are reported in Table 2. It is of note that the optimized structure of 1 is found of nearly *C*_2_ symmetry, with the two hydrides in equivalent μ_4_ co-ordination mode, with Rh–H (av.) = 1.664 Å and Ag–H (av.) = 2.074 Å. As in the experimental structure, the Ag atoms to which they are connected form an enlarged triangular face (Ag⋯Ag (av.) = 3.434 Å). Similar elongations are also found in the case of the hydride-containing icosahedron of 2 (Ag⋯Ag (av.) = 3.465 Å). Interestingly, whereas the SCXRD position of one of the hydrides in 2 is nearly perfectly μ_4_ (see above), it is intermediate between μ_4_ and μ_3_ in the DFT-optimized structure (Ag–H = 1.987, 2.098, and 2.376 Å). These results are consistent with a flat potential energy surface associated with the displacement of each hydride within its cage. As a whole, the CSM values follow the same trend as their experimental counterparts, but they are somewhat smaller, presumably due to the least steric hindrance of the model ligands.

**Table 2:** Selected computed data for compounds 1–3. Distances are given in Å, with corresponding Wiberg bond indices in brackets

Compound		1		2		3	
HOMO–LUMO gap (eV)		1.24		1.23		1.28	
Superatomic core		RhH@Ag_12_	RhH@Ag_12_	Pt@Ag_12_	PtH@Ag_12_	Pt@Ag_12_	Pt@Ag_12_
CSM		0.33	0.35	0.03	0.49	0.06	0.06
M_cent_–Ag_ico_ (av.)		2.877 [0.156]	2.876 [0.155]	2.848 [0.140]	2.903 [0.116]	2.851 [0.150]	2.851 [0.150]
Ag_ico_–Ag_ico_ (av.)		2.987 [0.070]	2.987 [0.070]	2.996 [0.075]	3.052 [0.068]	2.998 [0.081]	2.998 [0.081]
Ag_ico_–Ag_cap_ (av.)		3.070 [0.046]	3.064 [0.046]	3.097 [0.044]	3.123 [0.038]	3.092 [0.049]	3.092 [0.049]
M_center_–H		1.662 [0.251]	1.667 [0.246]	—	1.693 [0.224]	—	—
Ag–H (av.)		2.073 [0.103]	2.074 [0.104]	—	2.155 [0.097]	—	—
Natural atomic charges	M	−1.30	−1.29	−1.17	−0.94	−1.13	−1.13
	H	−0.30	−0.31	—	−0.34	—	—
	Ag_ico_ (av.)	0.25	0.25	0.20	0.27	0.21	0.21
	Ag_cap_ (av.)	0.57	0.57	0.58	0.58	0.57	0.57

With 33 Ag atoms and 17 formally anionic ligands, compound 3 has 33–17 = 16 so-called “free” electrons (of 5s(Ag) origin), which are responsible for the bonding within the bi-icosahedral core. We have shown previously^29^ that 3 is made of two weakly interacting vertex-sharing 8-electron icosahedral superatoms, with individual stable 1S^2^ 1P^6^ closed-shell configuration.^55^ In other words, 3 can be described as the supermolecular equivalent of the Ne_2_ van-der-Waals molecule.

We have also shown previously that in clusters containing an [(MH)@Ag_12_]^5+^ (M = Pd, Pt) core, the 1s(H) atomic orbital (AO) interacts principally with a d_z^2^_(M) orbital, creating an M–H 2-electron bond and leaving the (*n* + 1)s AO of M almost unperturbed, but now containing one electron.^4,7^

**Fig. 8:**
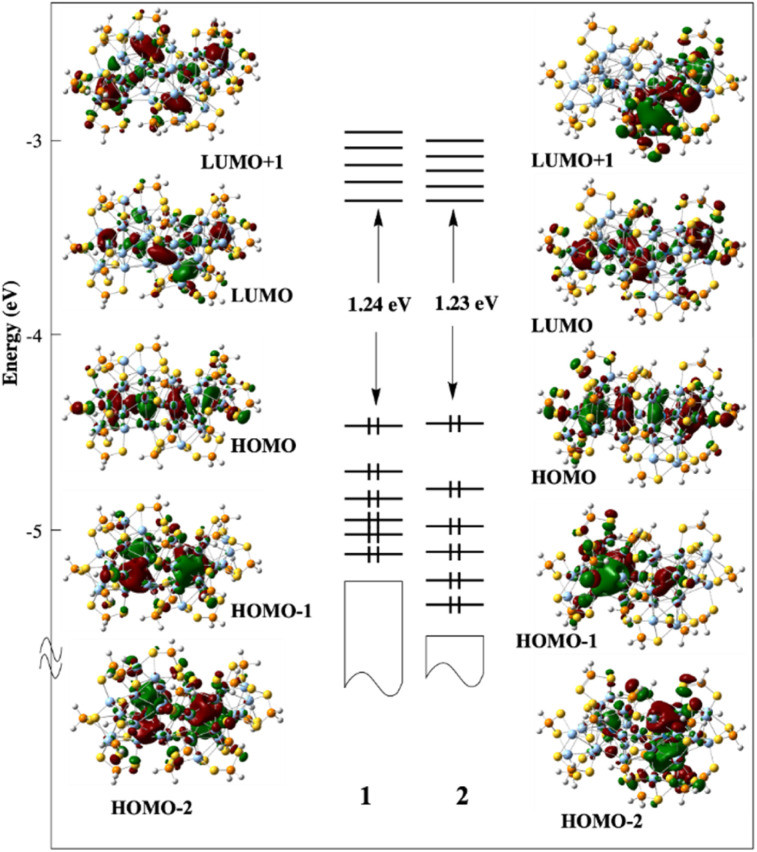
The Kohn–Sham frontier MO diagram of compounds 1 and 2. The hydride of 2 lies in the left-side icosahedron.

From the superatomic point of view, although the 1s(H) AO does not contribute significantly to the building of the superatomic orbitals, the hydride provides one electron to the *σ* (*n* + 1)s AO of the M atom, which in turn allows the cluster “free” electron count to achieve the closed-shell count of eight. This is what happens in 2, where PtH contributes with one electron; thus, 1 (PtH) + 32 (Ag) − 17 (ligands) = 16 = 2 × 8. The same reasoning holds for 1, where RhH is a zero-donor metal^1^ (“isoelectronic” to Pd), thus 33 (Ag) − 17 (ligands) = 16. Viewing the three NCs as 16-electron supermolecules is confirmed by the analysis of their electronic structure. In the three NCs, the 1S and 1P orbitals of the two icosahedra generate occupied combinations. The Kohn–Sham frontier orbital diagrams of 1 and 2 are shown in Fig. 8. Their compositions are provided in Fig. S23.† The HOMO of 1 and 2 is the out-of-phase combination of the icosahedra 1P_σ_ orbitals, whereas the HOMO−1 and HOMO−2 are the out-of-phase combinations of their 1P_π_ orbitals. The three lowest orbitals are their in-phase homologues, mixed with 4d(Ag_ico_) combinations. Similarly, the vacant 1D orbitals on the two icosahedra generate vacant combinations, the LUMO of the NCs being of 1D_σ_ character. The participation of the hydrides in the superatomic combinations is negligible (Fig. S23†). Nevertheless, the M–H bonding is relatively strong, as indicated by the corresponding Wiberg bond indices (Table 2), which are substantially larger than their Ag–H counterparts. These features, as well as the computed natural atomic charges (Table 2), are typical for 8-electron superatoms containing or not containing encapsulated hydrogens.^4^ The time-dependent-DFT (TD-DFT) simulated UV-vis spectra of 1 and 2 are shown in Fig. S24.† They are quite similar and qualitatively agree with their experimental counterparts (Fig. 6).

For both compounds, the band (or shoulder) of the lowest energy (∼660 and ∼670 nm for 1 and 2, respectively) can be identified as of 1P → 1D nature. The next band (∼460 nm for both compounds) is also of 1P → 1D character, but with 4d(Ag_ico_) → 1D ad-mixture. The band of higher energy is of MLCT nature.

## Conclusion

The controlled isolation of two novel hydride-containing 16-electron supermolecules, (RhH)_2_Ag_33_[S_2_P(OPr)_2_]_17_ (1), and PtHPtAg_32_[S_2_P(OPr)_2_]_17_ (2), was successful. Additionally, the interstitial hydride atom in compound 2 can be removed by thermal treatment to form Pt_2_Ag_33_[S_2_P(OPr)_2_]_17_ (3). Significant structural distortions can be found in the hydride-doped icosahedra. Notably, while compound 1 is the first example of a group 9-doped supermolecule, both compounds 1 and 2 are the first hydride-containing 16-electron superatomic molecules. Importantly, DPV and absorption spectroscopy measurements have confirmed that the assembly of superatoms into supermolecules leads to a significant decrease in the HOMO–LUMO gap. All NCs show strong photoluminescence in the near-infrared region at 77 K in 2-MeTHF glass. This work will provide inspiration for hydride-containing superatomic molecules, and it is expected that more complex hydride-encapsulating supermolecular architectures with potentially unprecedented properties will be synthesized in the future.

## Data availability

Crystallographic data for 1–3 has been deposited at the Cambridge Crystallographic Data Centre (CCDC) under 2342395, 2342396, and 2342397, and can be obtained from https://www.ccdc.cam.ac.uk/structures/.

## Author contributions

T.-H. C., M. N. P., investigation, data curation, formal analysis, methodology, writing. Y.-Y. W., Y. N., Y. N., J.-Y. C., Y. J. C., S. K. data curation, formal analysis. J.-Y. S. data curation, formal analysis, writing. C. W. L. supervision, writing, conceptualization, project administration, resources.

## Conflicts of interest

There are no conflicts to declare.

## Supplementary Material

SC-OLF-D4SC02920H-s001

SC-OLF-D4SC02920H-s002
