# Supplementary material for: Controlled aggregation of Pt/PtH/Rh/RhH doped silver superatomic nanoclusters into 16-electron supermolecules
Source: Chem Sci. 2024 Aug 22;15(36):14660–7. doi: 10.1039/d4sc02920h (PMC11376050; doi:10.1039/d4sc02920h)
Supplement: SC-015-D4SC02920H-s001 [file SC-015-D4SC02920H-s001.pdf]

Supplementary Information (SI) for Chemical Science.
This journal is © The Royal Society of Chemistry 2024

Supplementary Information

Controlled Aggregation of Pt/PtH/Rh/RhH doped Silver Superatomic Nanoclusters into 16-electron Supermolecules

Tzu-Hao Chiu,[a] Michael N. Pillay,[a] Ying-Yann Wu,[a] Yoshiki Niihori,[b] Yuichi Negishi,[b] Jie-Ying Chen,[c] Yuan Jang Chen,[c] Samia Kahlal,[d] Jean-Yves Saillard,[d*] and C. W. Liu[a*]

a. Department of Chemistry, National Dong Hwa University, Hualien 97401, Taiwan (Republic of China).

b. Department of Applied Chemistry, Tokyo University of Science, 1-3 Kagurazaka, Shinjuku, Tokyo 162-8601, Japan.

c. Department of Chemistry, Fu Jen Catholic University, New Taipei City, 24205, Taiwan (Republic of China).

d. Univ Rennes, CNRS, ISCR-UMR 6226, F-35000 Rennes, France.* Correspondence: E-mail: chenwei@mail.ndhu.edu.tw; Tel: +886-3-890-3607; Fax : +886-3-890-0162; http://faculty.ndhu.edu.tw/~cwl/index.htm

Experimental Section

1.1 General remarks

All chemicals were purchased from commercial sources and used as received. Solvents were purified following standard protocols. All reactions were carried out under N_2 atmosphere by using standard Schlenk techniques. $[Ag(CH_3CN)_4]PF_6$ and $NH_4[S_2P(O^nPr)_2]$ were prepared by following the procedure reported in literature.[S1,S2] NMR spectra were recorded on a Bruker AVII-400 and AV-600 MHz NMR spectrometers. The chemical shift (δ) is reported in ppm and Hz, respectively. ESI-mass spectra recorded on a Bruker maXis Q-TOF mass spectrometer (Bruker Daltonik GmbH, Germany). UV–visible-NIR absorption spectra were measured on a Shimadzu UV3101PC spectrophotometer at 298 K, using quartz cells with a path length of 1 cm. Luminescence spectra and lifetime were recorded on an Edinburgh FLS920 fluorescence spectrometer. PLQY study was conducted on a HORIBA JOBIN YVON iHR 550 spectrometer, equipped with a HORIBA Symphony InGaAs-1700 (for the NIR) detector head mounted on the exit port. $[Ru(bpy)_2(en)]^{2+}$ (bpy = bpyridine; en = ethylenediamine) was the reference for the determination at 87 K.

1.2 Synthesis

1.2.1 Synthesis of [$(RhH)_2Ag_{33}\{S_2P(O^nPr)_2\}_{17}$] (1)

In a Flame-dried Schlenk tube, [$Ag(CH_3CN)_4$]PF_6 (0.50 g, 0.86 mmol) and NH_4[$S_2P(O^nPr)_2$] (0.14 g, 0.43 mmol) were dissolved in THF, and kept stirring at -20℃ for 5 minutes. [Rh(COD)Cl]$_2$ (0.02 g, 0.043 mmol) was added to the above solution. After 10 minutes, $NaBH_4$ (0.02 g, 0.86 mmol) was added, and kept stirring for 12 hours. The reaction mixture was dried under reduced pressure. The residue was washed with DCM/DI-Water. The DCM layer was dried under reduced pressure and subsequently purified by thin-layer chromatography, with MeOH as a mobile phase. Finally, a gray-black product can be obtained. The yield of **1** is 10% based on Ag.

[$(RhH)_2Ag_{33}\{S_2P(O^nPr)_2\}_{17}$] **(1)**

$^{31}P\{^1H\}$ NMR (161.97 MHz, $CDCl_3$, δ, ppm, r.t.): 104.7 (br, 10 S_2P), 101.0 (s, 2 S_2P), 96.9 (br, 5 S_2P). ^1H NMR (400 MHz, $CDCl_3$, δ, ppm, r.t.): -12.1 (br, μ_4-H, 2H) 0.9-1.0 (m, CH_3, 102H), 1.67-1.89 (m, CH_2, 68H), 3.97 (q, OCH_2, 8H), 4.12 (q, OCH_2, 40H), 4.34 (q, OCH_2, 20H). ESI-MS (*m/z*): exp. 7392.983 (calc. for $[M + H]^+$: 7392.990). UV-vis [λ_{max} in nm, (ε in $M^{-1}cm^{-1}$)]: 380 (65500), 500 (35500), 708 (6000).

1.2.1 Synthesis of [$PtHPtAg_{32}\{S_2P(O^nPr)_2\}_{17}$] (2) and [$Pt_2Ag_{33}\{S_2P(O^nPr)_2\}_{17}$] (3)

In a Flame-dried Schlenk tube, [$Ag(CH_3CN)_4$]PF_6 (0.50 g, 0.86 mmol) and NH_4[$S_2P(O^nPr)_2$] (0.14 g, 0.43 mmol) were dissolved in THF, and kept stirring at -20℃ for 5 minutes. Pt[$S_2P(O^nPr)_2$]$_2$ (0.02 g, 0.043 mmol) was added to the above solution. After 10 minutes, $NaBH_4$ (0.02 g, 0.86 mmol) was added, and kept stirring for 12 hours. The reaction mixture was dried under reduced pressure. The residue was washed with DCM/DI-Water. The DCM layer was dried under reduced pressure and further purified by thin-layer chromatography with MeOH as a mobile phase. The first brown band is [$PtAg_{20}\{S_2P(O^nPr)_2\}_{12}$]. The second purple band is [$PtHPtAg_{32}\{S_2P(O^nPr)_2\}_{17}$] (**2**). The final purple band is [$Pt_2Ag_{33}\{S_2P(O^nPr)_2\}_{17}$] (**3**) (Figure S22). The yields of **2**, and **3** are 2% and 11%, respectively, based on Ag.

[$PtHPtAg_{32}\{S_2P(O^nPr)_2\}_{17}$] **(2)**

$^{31}P\{^1H\}$ NMR (161.97 MHz, $CDCl_3$, δ, ppm, r.t.): 106.0 (br, 5 S_2P), 104.8 (br, 5 S_2P), 101.0 (s, 2 S_2P), 96.2 (br, 5 S_2P). ^1H NMR (400 MHz, $CDCl_3$, δ, ppm, r.t.): -10.4 (br, $^1J_{1H\text{-}195Pt}$ = 512Hz at 293K) 0.9-1.0 (m, CH_3, 102H), 1.63-1.87 (m, CH_2, 68H), 3.99 (q, OCH_2, 8H), 4.12 (q, OCH_2, 40H), 4.31 (q, OCH_2, 20H). ESI-MS (*m/z*): exp. 3841.954 (calc. for $[M + 2Ag]^{2+}$: 3841.987). UV-vis [λ_{max} in nm, (ε in $M^{-1}cm^{-1}$)]: 490 (26000), 700 (4000).

1.3 X-ray crystallography

Single crystals suitable for X-ray diffraction analysis of **1**, **2**, and **3** was obtained by evaporating MeOH solution at 4°C within a week. The single crystals were mounted on the tip of glass fiber coated in paratone oil, then frozen. Data were collected on a Bruker APEX II CCD diffractometer using graphite monochromated Mo $K\alpha$ radiation (λ = 0.71073 Å) at 100 K. Absorption corrections for area detector were performed with SADABS[S3] and the integration of raw data frame was performed with SAINT.[S4] The structure was solved by direct methods and refined by least-squares against F^2 using the SHELXL-2018/3 package,[S5,S6] incorporated in SHELXTL/PC V6.14.[S7] All non-hydrogen atoms were refined anisotropically. CCDC 2342395 (**1**), 2342396 (**2**) and 2342397 (**3**) contains the supplementary crystallographic data for compounds **1-3** in this article. These data can be obtained free of charge from the Cambridge Crystallographic Data Centre via www. ccdc.cam.ac.uk/data_request/cif.

1.4 Computational Details

Geometry optimizations were performed by density functional theory (DFT) calculations with the Gaussian 16 package,[s8] using the BP86 functional[s9] and the all-electron Def2-TZVP set from EMSL Basis Set Exchange Library.[s10] All the optimized geometries were characterized as true minima by vibrational analysis. The NAO charges and Wiberg bond indices were computed with the NBO 6.0 program[s11] on single-point calculations performed with the BP86 functional and the Def2-SVP basis set for computational limitations.[s12] The UV-visible transitions were calculated by means of time-dependent DFT (TD-DFT) calculations, with the CAM-B3LYP functional[s13] and the Def2-TZVP basis set. Only singlet-singlet, *i.e.* spin-allowed, transitions have been computed. The UV-visible spectra were simulated from the computed TD-DFT transitions and their oscillator strengths by using the Multiwfn program,[s14] each transition is associated with a Gaussian function of half-height width equal to 2000 cm^{-1}. The compositions of the molecular orbitals were calculated using the AOMix program.[s15]

References

S1. A. A. M. Alyl, B. Walfortn, H. Z. Lang, *Kristallogr. NCS* **2004**, *219*, 489-491.

S2. P. Wystrach, E. O. Hook, G. L. M. Christopher, *J. Org. Chem.* **1956**, *21*, 705-705.

S3. SADABS, version 2014-11.0, Bruker Area Detector Absorption Corrextions (Bruker AXS Inc., Madison, WI, 2014).

S4. SAINT, included in G. Jogl, V4.043: Software for the CCD detector system (Bruker Analytical: Madison, WI, 1995).

S5. G. M. Sheldrick, *Acta Cryst. A* **2008**, *64*, 112-122.

S6. T. Gruene, H. W. Hahn, A. V. Luebben, F. Meilleur, G. M. J. Sheldrick, *Appl. Cryst.* **2014**, *47*, 462-466.

S7. SHELXTL, version 6.14. (Bruker AXS Inc., Madison, Wisconsin, USA, 2003).

S8. M. J. Frisch, G. W. Trucks, H. B. Schlegel, G. E. Scuseria, M. A. Robb, J. R. Cheeseman, G. Scalmani, V. Barone, G. A. Petersson, H. Nakatsuji, X. Li, M. Caricato, A. V. Marenich, J. Bloino, B. G. Janesko, R. Gomperts, B. Mennucci, H. P. Hratchian, J. V. Ortiz, A. F. Izmaylov, J. L. Sonnenberg, D. Williams-Young, F. Ding, F. Lipparini, F. Egidi, J. Goings, B. Peng, A. Petrone, T. Henderson, D. Ranasinghe, V. G. Zakrzewski, J. Gao, N. Rega, G. Zheng, W. Liang, M. Hada, M. Ehara, K. Toyota, R. Fukuda, J. Hasegawa, M. Ishida, T. Nakajima, Y. Honda, O. Kitao, H. Nakai, T. Vreven, K. Throssell, Jr. J. A. Montgomery, J. E. Peralta, F. Ogliaro, M. J. Bearpark, J. J. Heyd, E. N. Brothers, K. N. Kudin, V. N. Staroverov, T. A. Keith, R. Kobayashi, J. Normand, K. Raghavachari, A. P. Rendell, J. C. Burant, S. S. Iyengar, J. Tomasi, M. Cossi, J. M. Millam, M. Klene, C. Adamo, R. Cammi, J. W. Ochterski, R. L. Martin, K. Morokuma, O. Farkas, J. B. Foresman, D. J. Fox, Gaussian, Inc., Wallingford CT, **2016**; Gaussian 16, Revision A.03.

S9. a) A. D. Becke, *Phys. Rev. A*, **1988**, *38*, 3098-3100; b) J. P. Perdew, *Phys. Rev. B*, **1986**, *33*, 8822-8824.

S10. a) A. Schaefer, H. Horn, R. Ahlrichs, *J. Chem. Phys.* **1992**, *97,* 2571-2577; b) A. Schaefer, C. Huber, R. Ahlrichs, *J. Chem. Phys.* **1994**, *100*, 5829-5835.

S11. a) E. D. Glendening, C. R. Landis, F. Weinhold, *J. Comput. Chem.*, **2013**, *34*, 1429–1437. b) E. D. Glendening, J. K. Badenhoop, A. E. Reed, J. E. Carpenter, J. A. Bohmann, C. M. Morales, C. R. Landis, F. Weinhold, *NBO 6.0*; Theoretical Chemisty Institute, University of Wisconsin, Madison, WI, **2013,** http://nbo6.chem.wisc.edu.

S12. a) F. Weigend, R. Ahlrichs, *Phys. Chem. Chem. Phys*. **2005**, *7*, 3297-3305; b) F. Weigend, *Phys. Chem. Chem. Phys.* **2006**, *8,* 1057-1065.

S13. T. Yanai , D. Tew, N. Handy, *Chem. Phys. Lett.* **2004**, *393*, 51-57.

S14. T. Liu, F. Chen, *J. Comput. Chem*. **2012**, *33*, 580–592.

S15. Gorelsky, S. I. AOMix program, http://www.sg-chem.net.

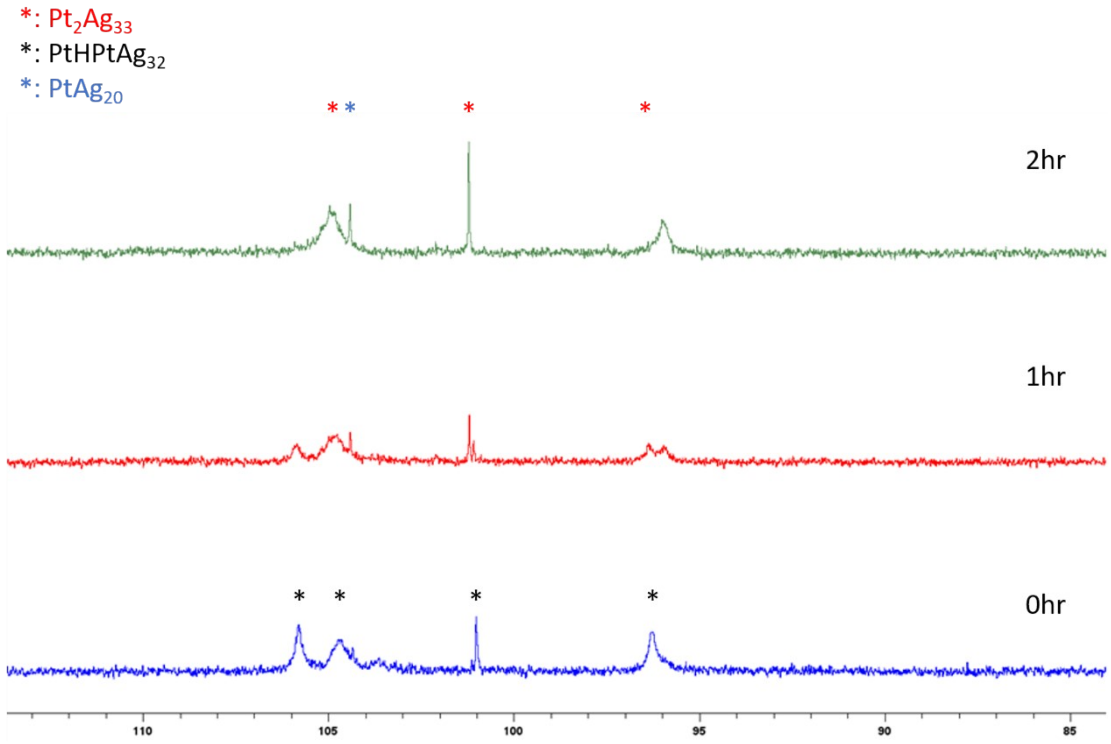

Figure S1. Time-dependent ^{31}P NMR spectra ($CDCl_3$) of **2** under 60°C.

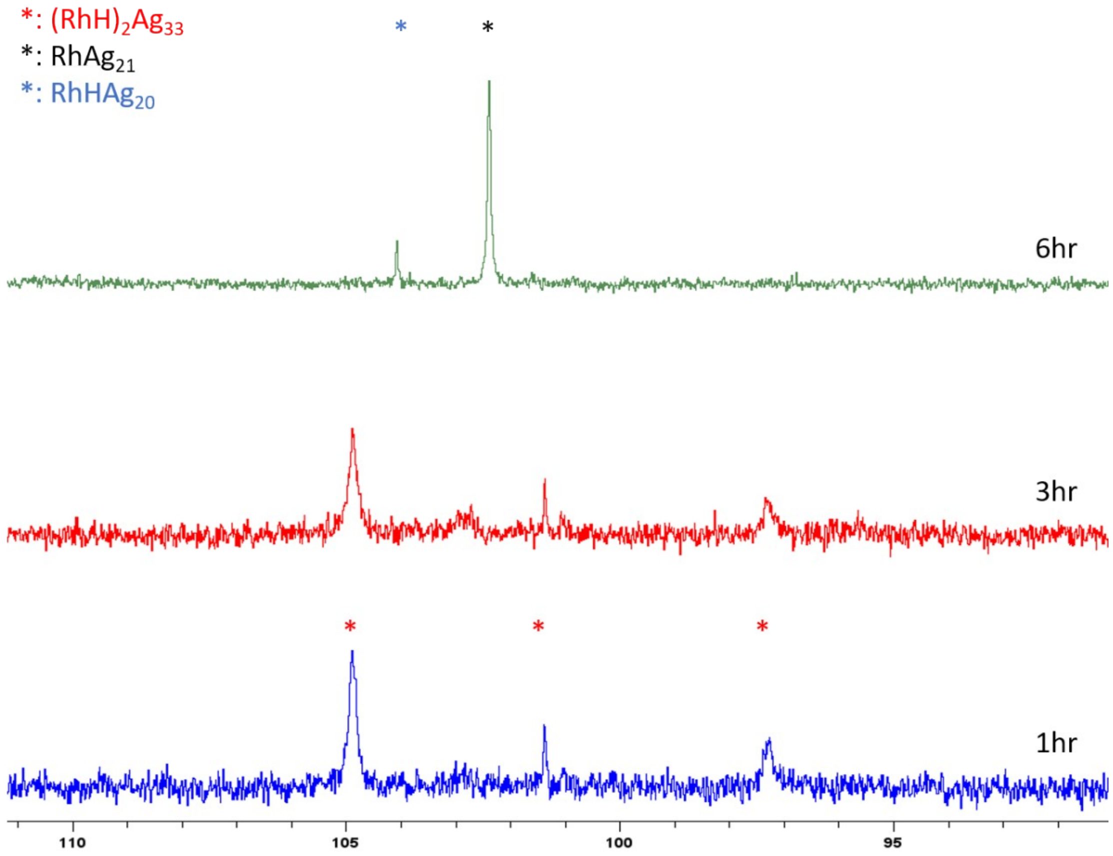

Figure S2. Time-dependent ^{31}P NMR spectra ($CDCl_3$) of **1** under 60°C.

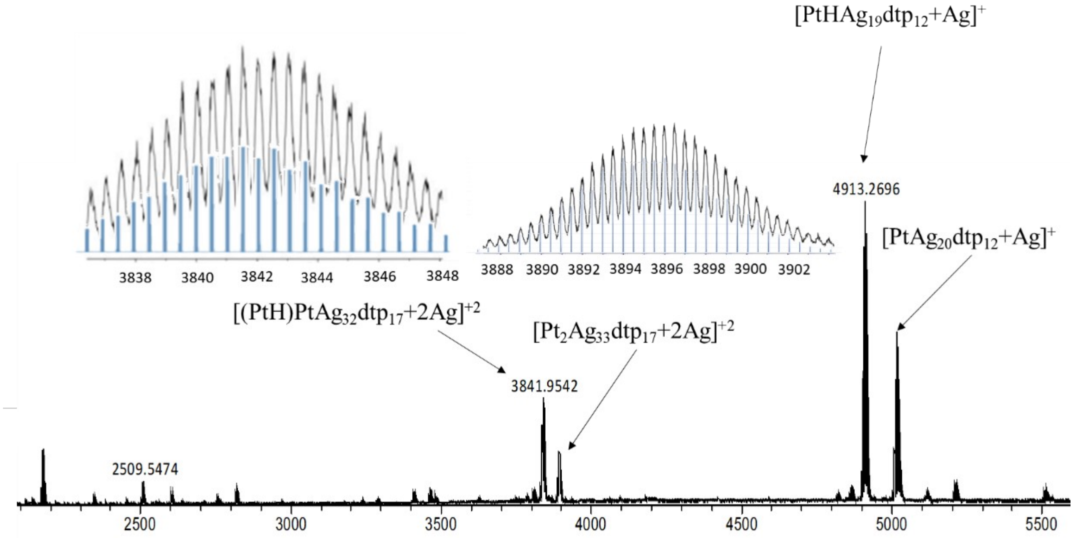

Figure S3. Positive-mode ESI mass spectrum of **2**. Insets: experimental (black) and simulated (blue).

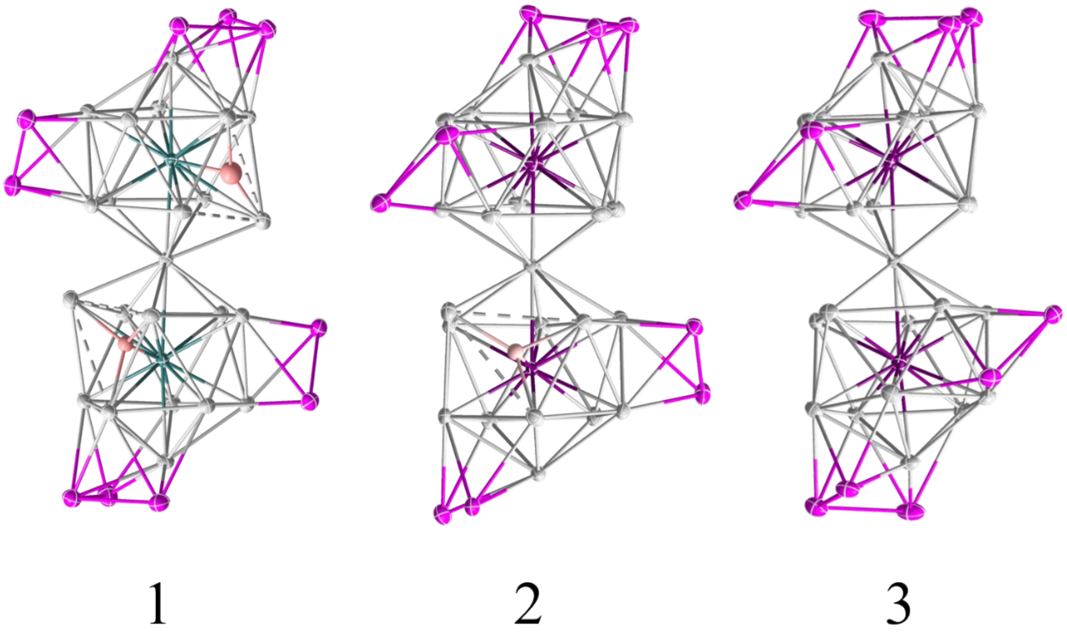

Figure S4. Metal framework of **1**-**3**.

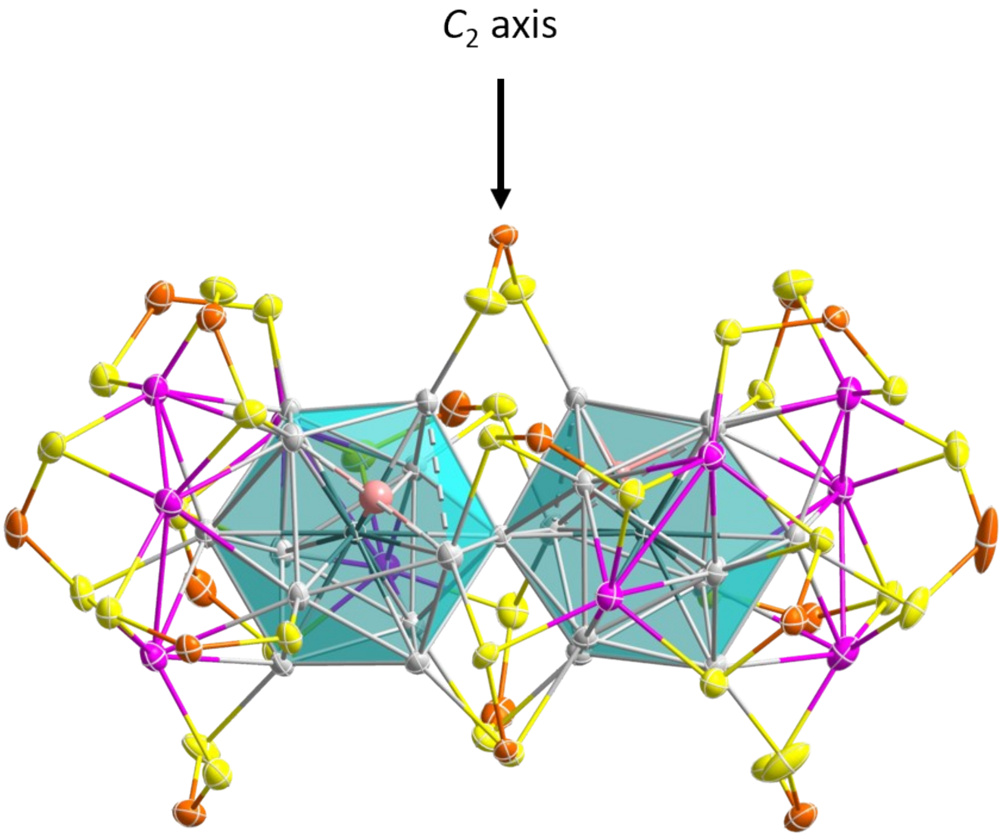

Figure S5. Side view of **1.**

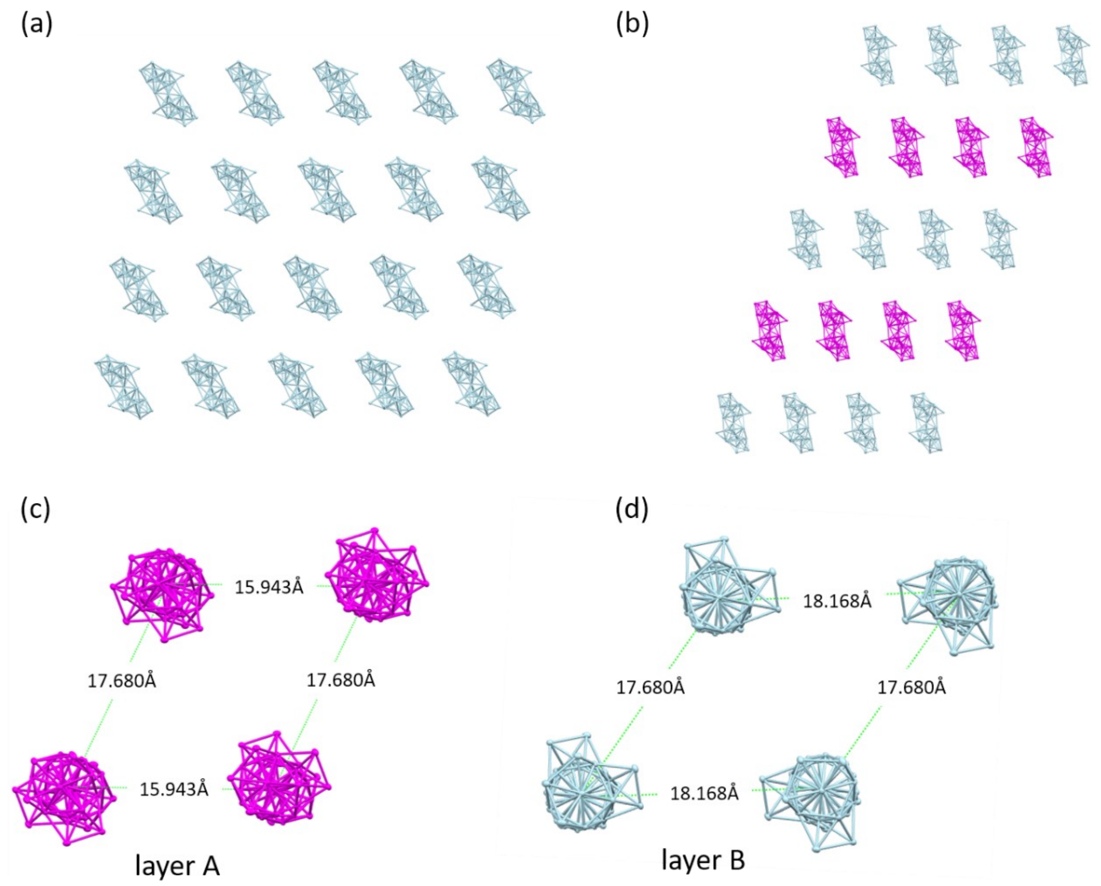

Figure S6. (a)Packing diagram of previously reported Pt_2Ag_{33}(b) packing diagram of **3** (c) layer A (d) layer B.

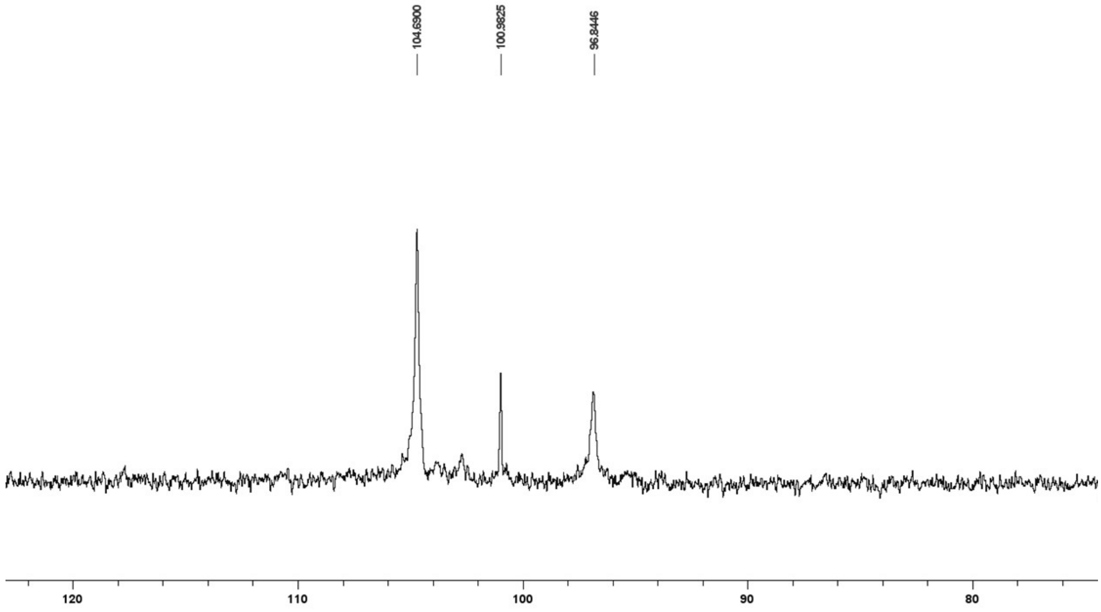

Figure S7. ^{31}P NMR spectrum ($CDCl_3$) of **1**.

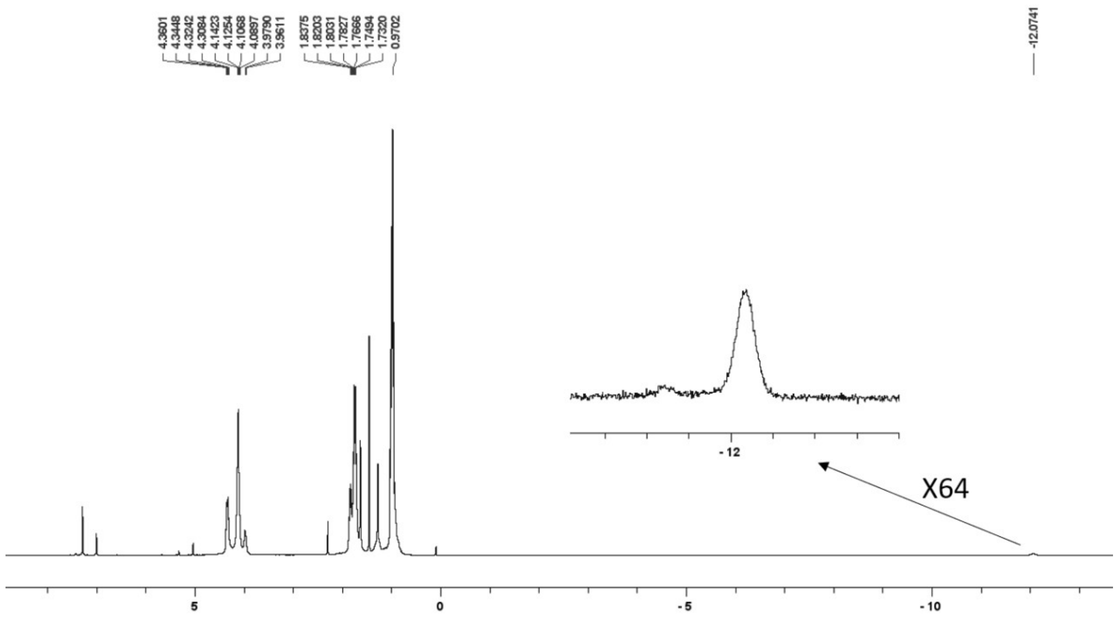

Figure S8. ^{1}H NMR spectrum ($CDCl_3$) of **1**.

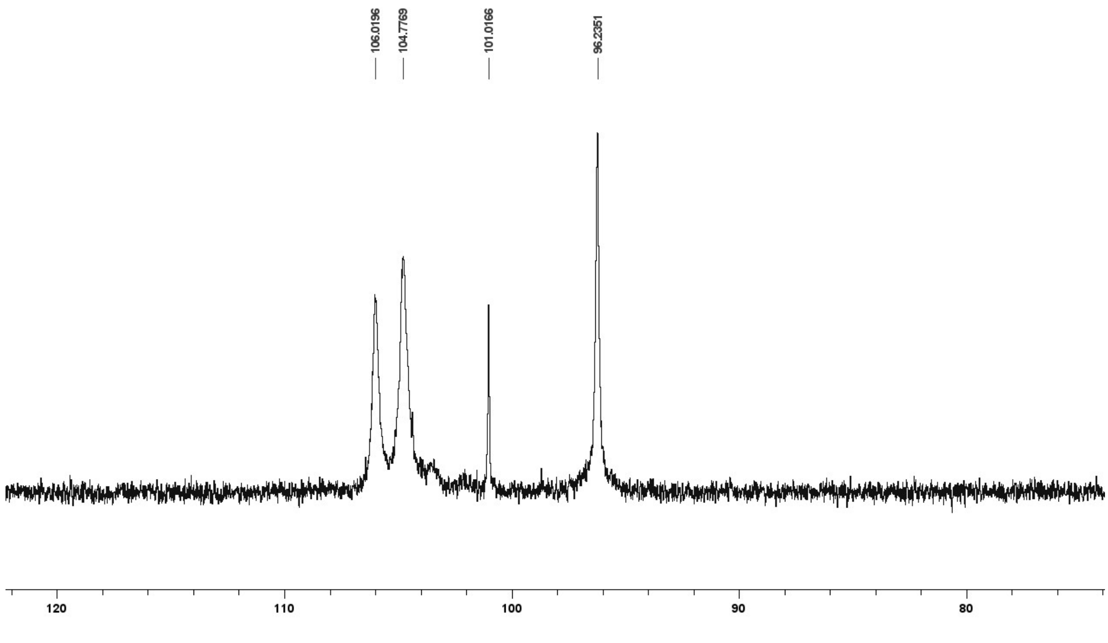

Figure S9. ^{31}P NMR spectrum ($CDCl_3$) of **2**.

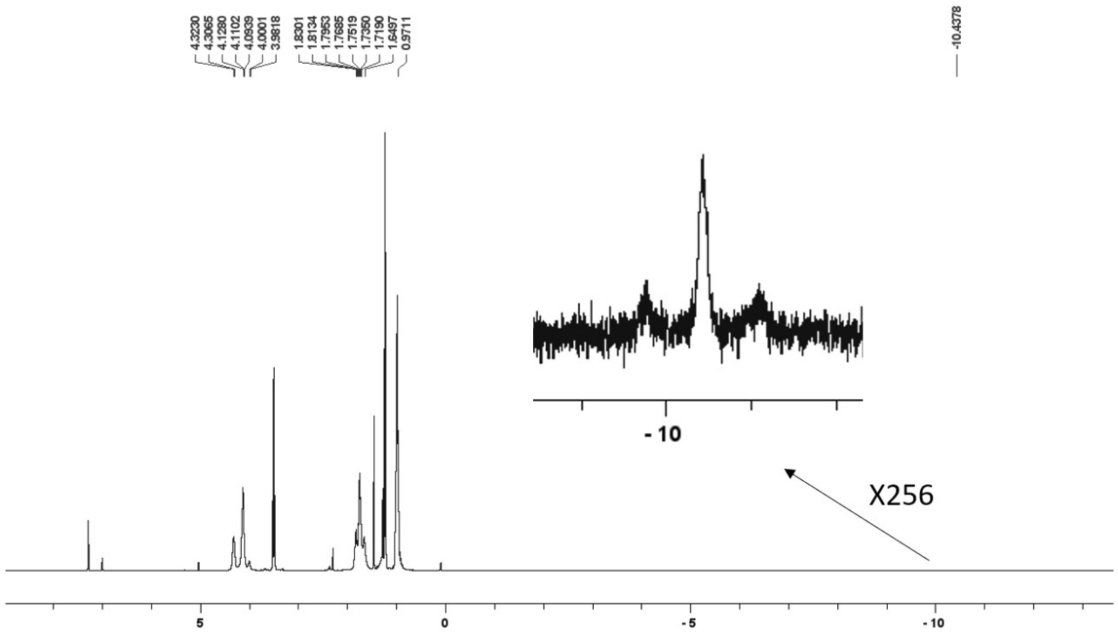

Figure S10. ^{1}H NMR spectrum ($CDCl_3$) of **2**.

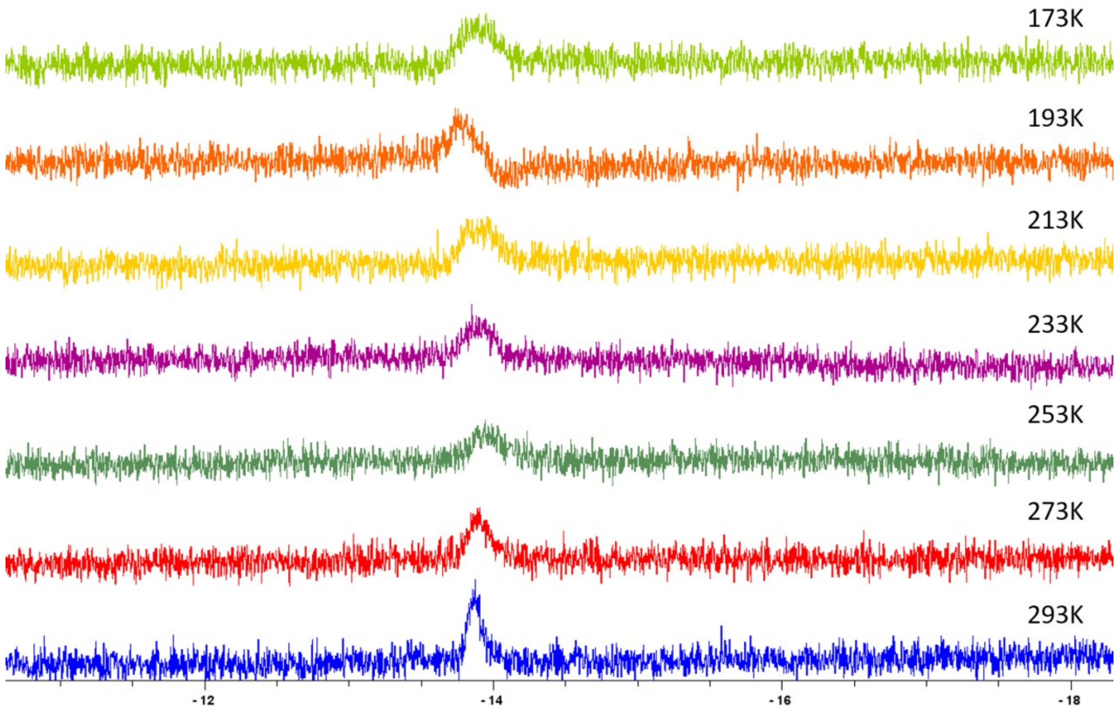

Figure S11. VT ^{1}H NMR spectrum (THF-d8) of **1**.

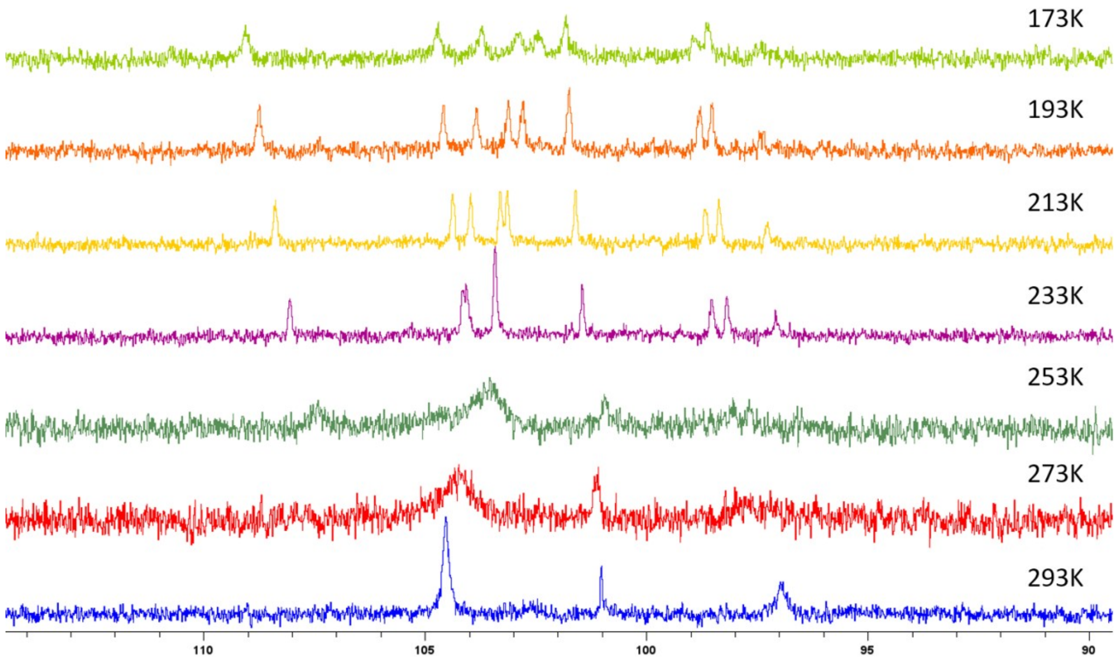

Figure S12. VT ^{31}P NMR spectrum (THF-d8) of **1**.

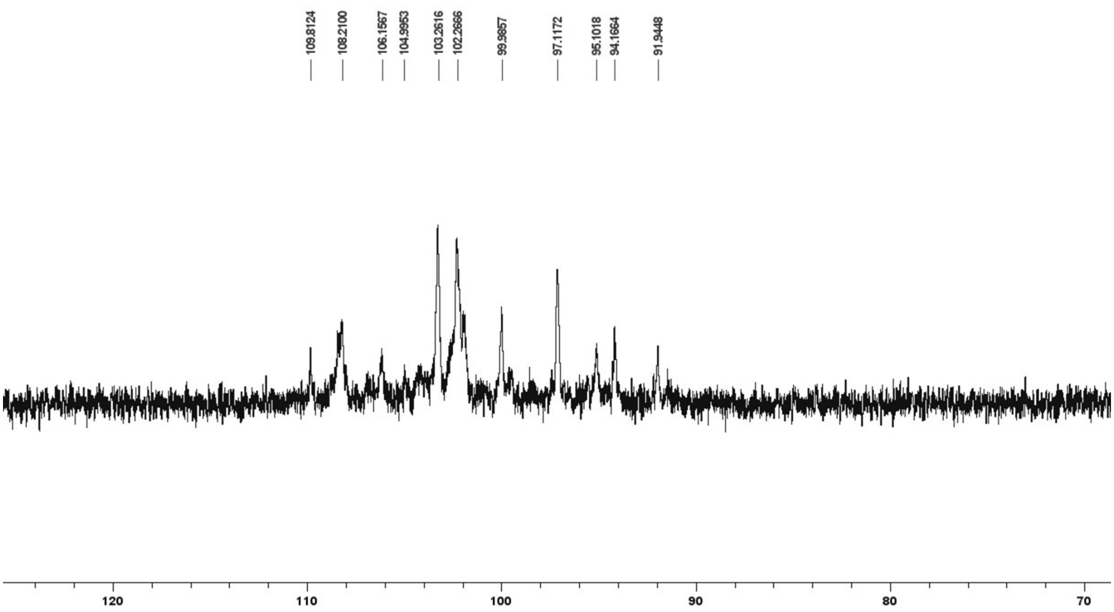

Figure S13. ^{31}P NMR spectrum (THF-d8) of **2** at 173K.

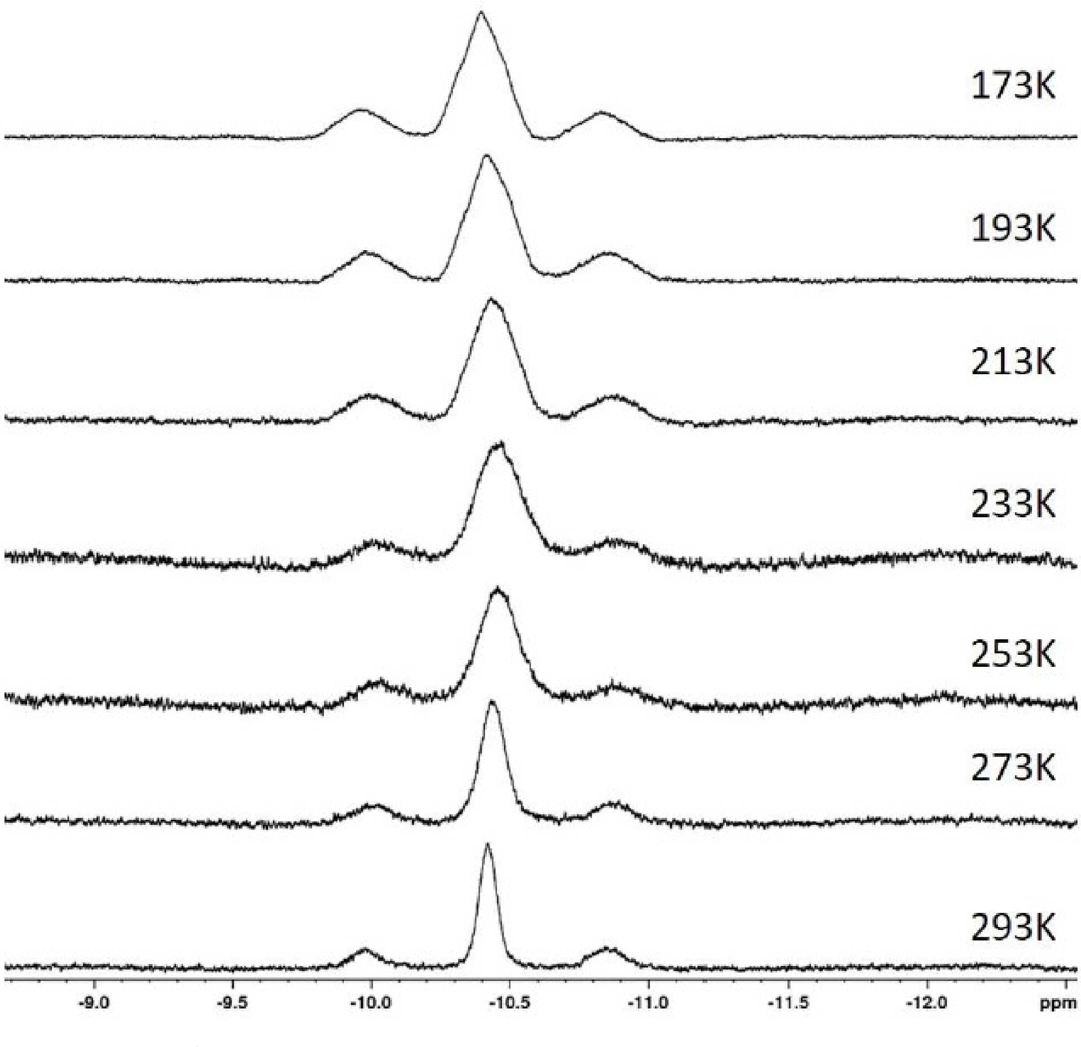

Figure S14. VT ^{1}H NMR spectrum (THF-d8) of **2**.

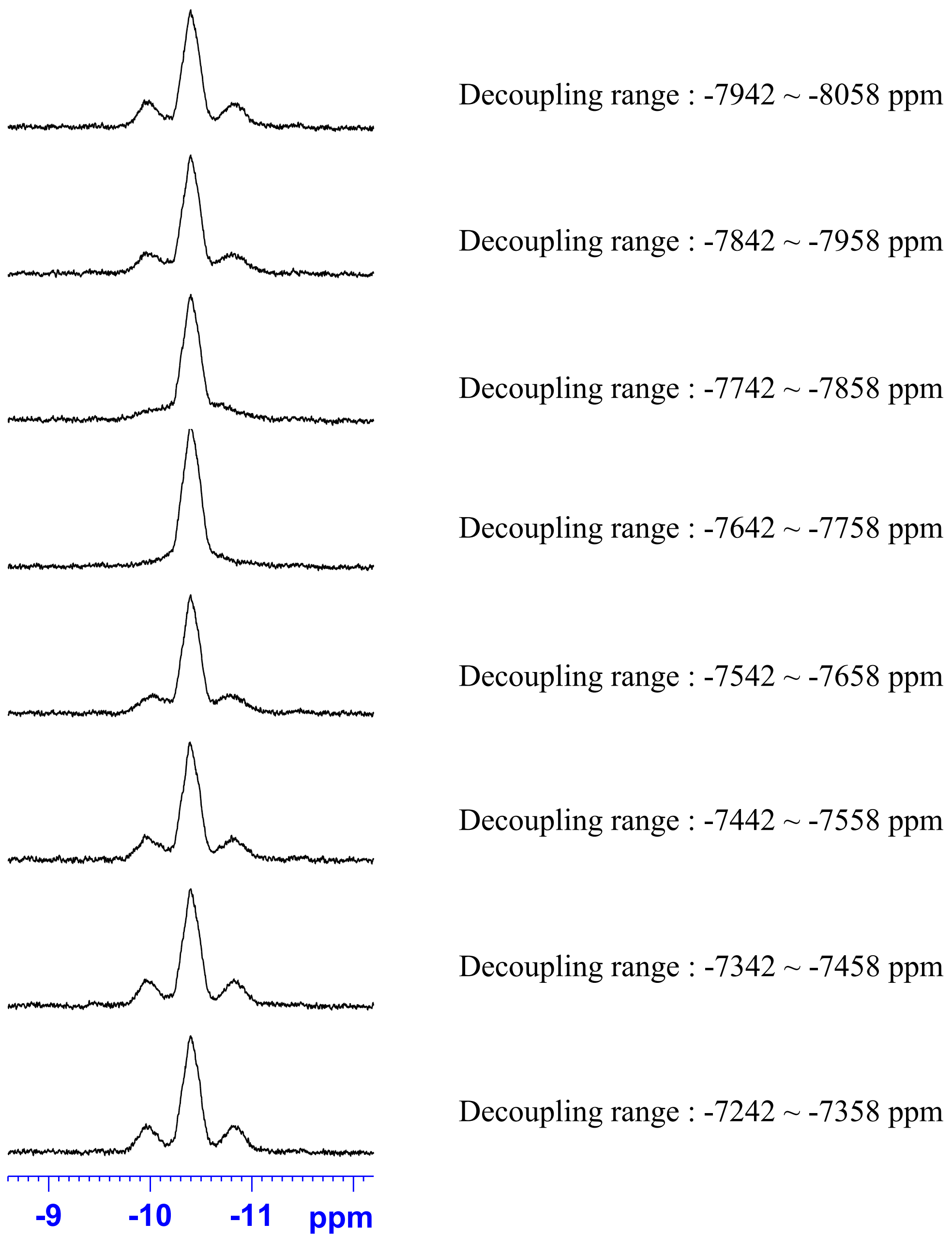

Figure S15. $^{1}H\{^{195}Pt\}$ NMR spectra of **2** at 173 K with various ^{195}Pt decoupling ranges.

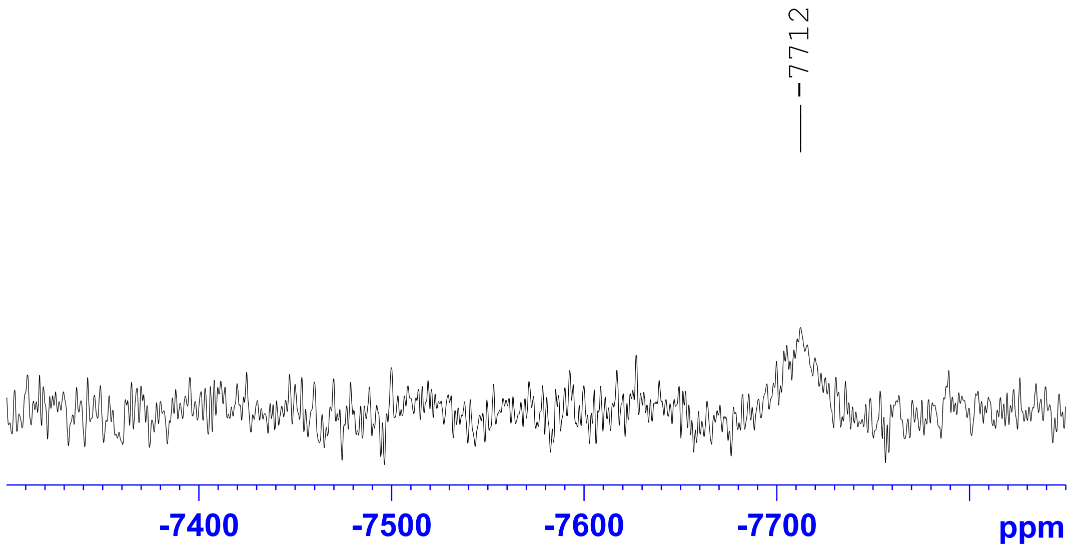

Figure S16. The $^{195}Pt\{^{1}H\}$ spectrum of **2** at 173K.

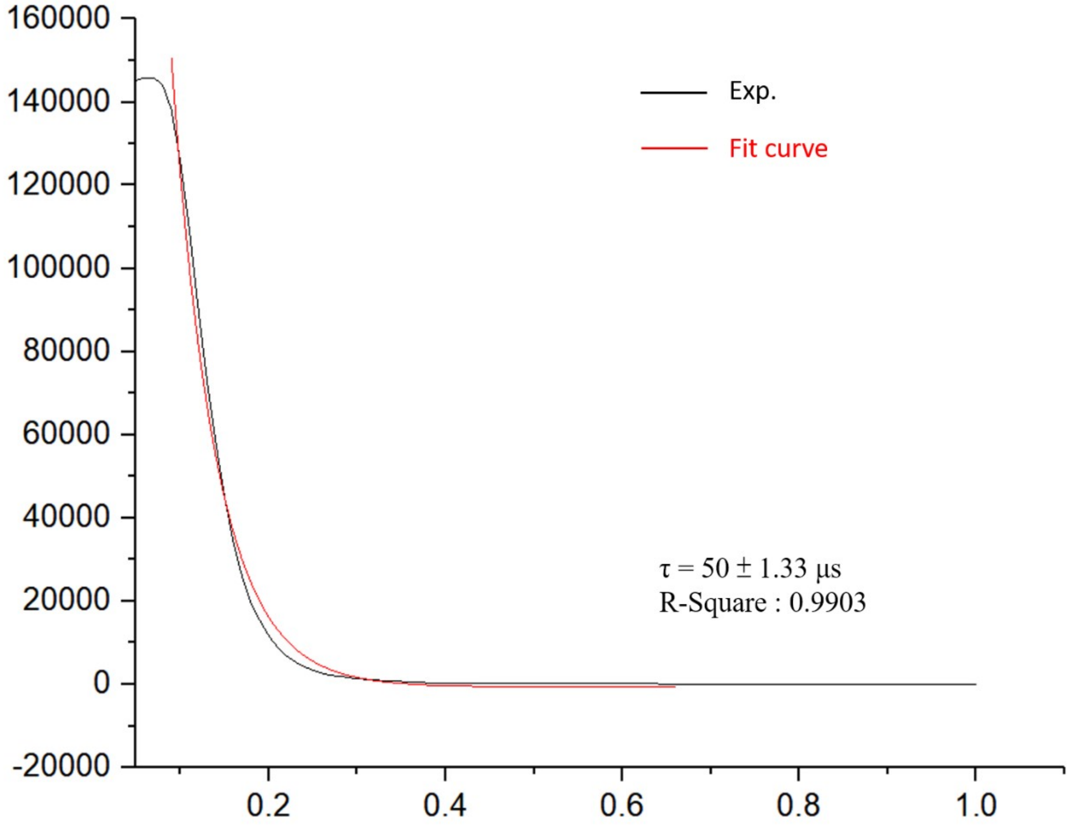

Figure S17. Time-resolved photoluminescence spectrum of **1**.

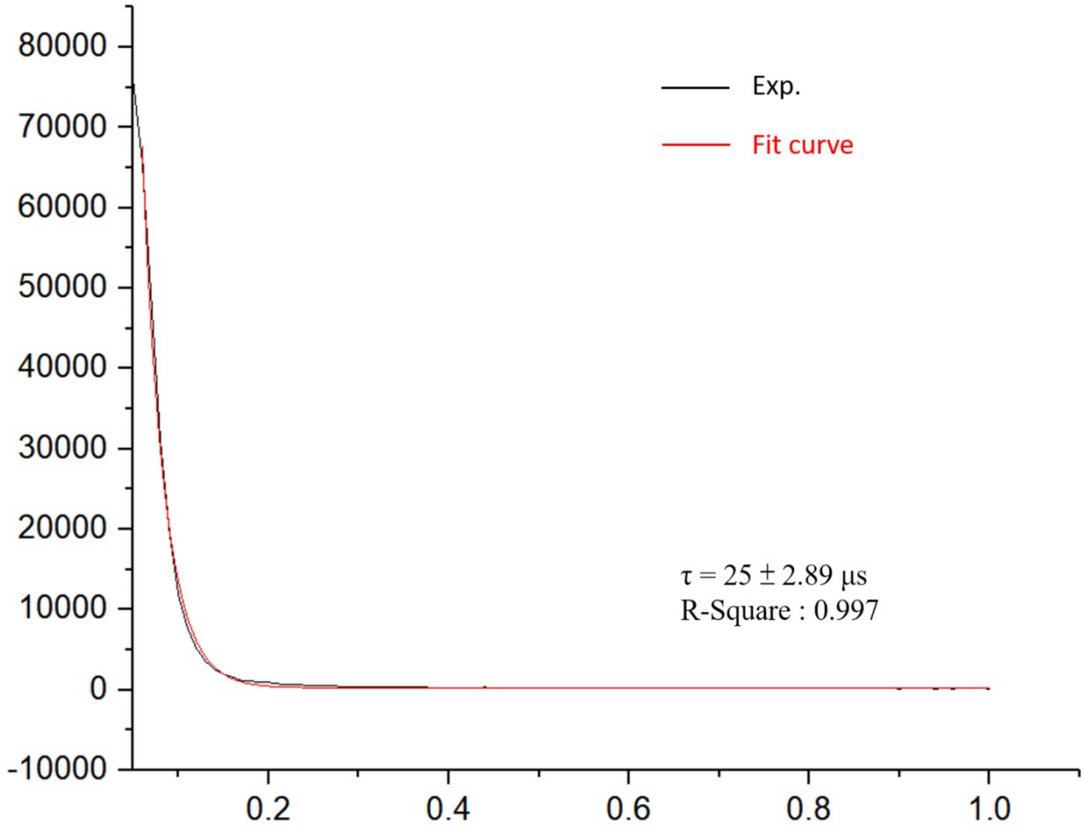

Figure S18. Time-resolved photoluminescence spectrum of **2**.

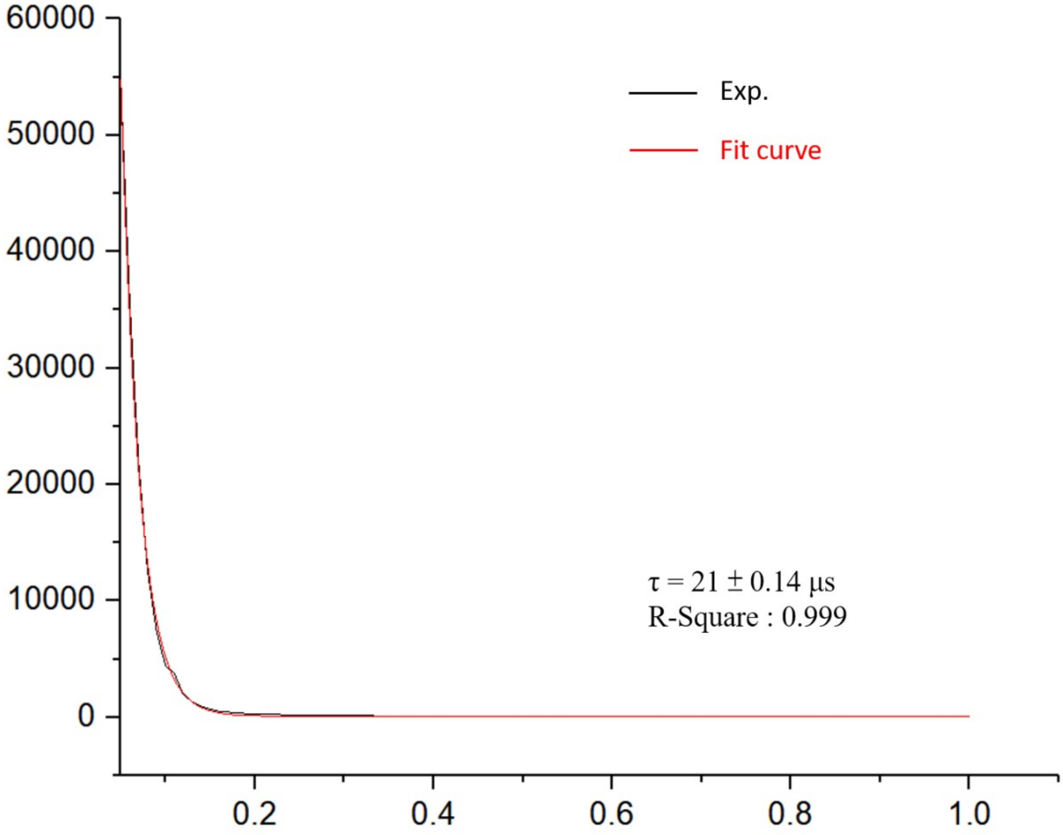

Figure S19. Time-resolved photoluminescence spectrum of **3**.

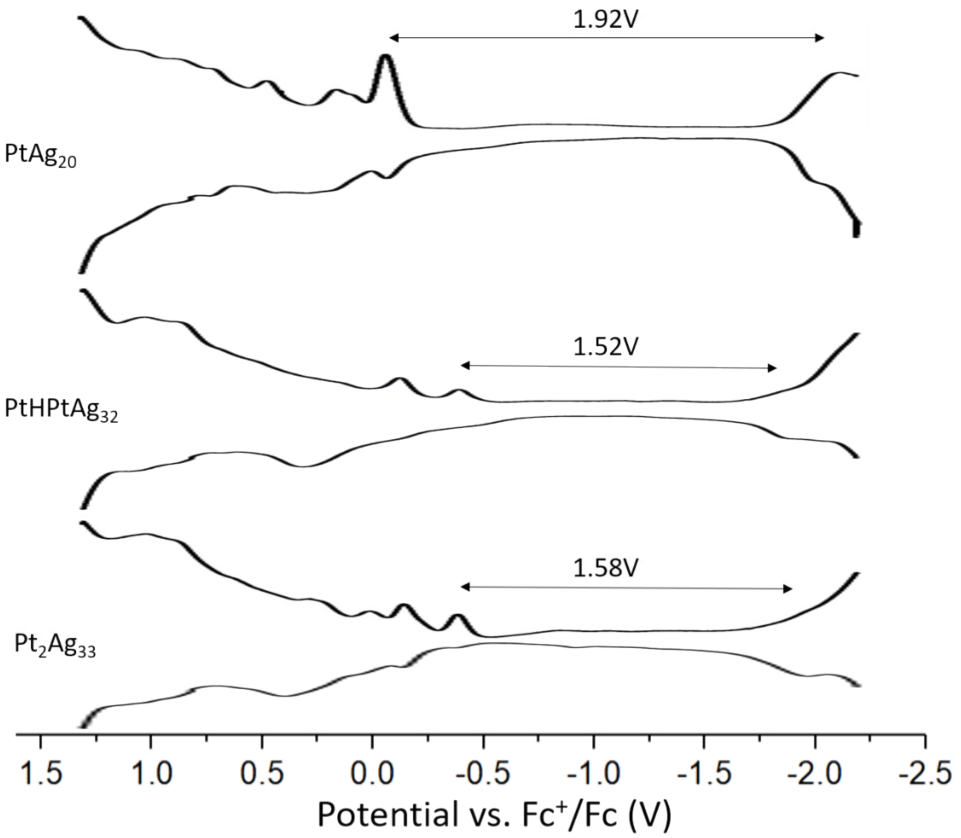

Figure S20. Differential pulse voltammograms of $PtAg_{20}[S_2P(OPr)_2]_{12}$, $PtHPtAg_{32}[S_2P(OPr)_2]_{17}$ and $Pt_2Ag_{33}[S_2P(OPr)_2]_{17}$.

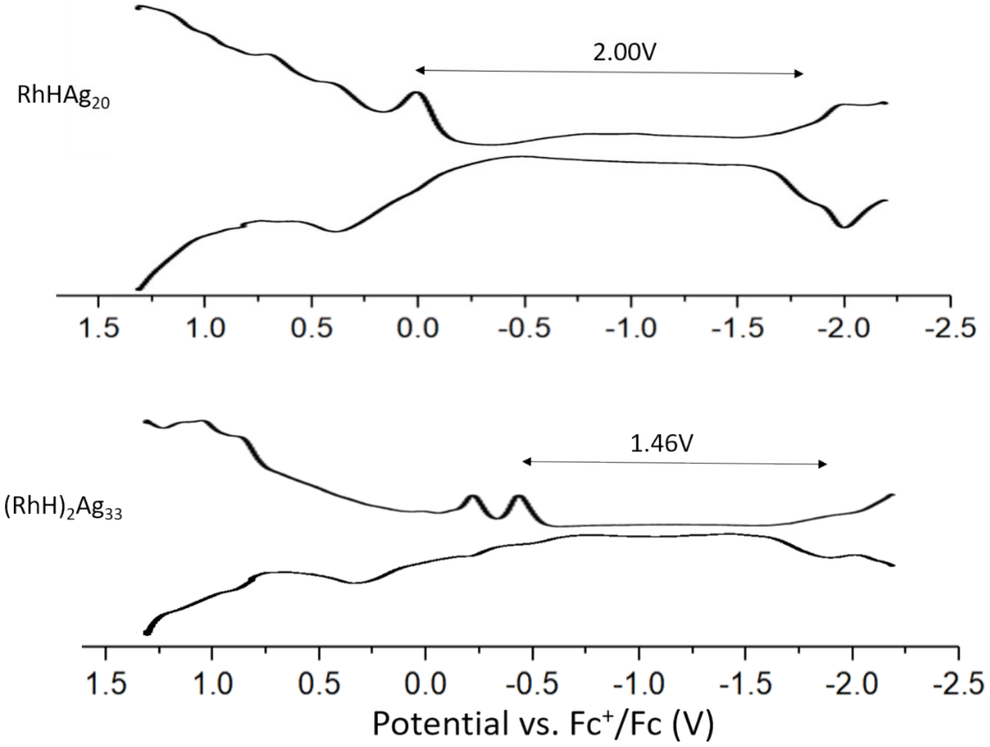

Figure S21. Differential pulse voltammograms of $RhHAg_{20}[S_2P(OPr)_2]_{12}$ and $(RhH)_2Ag_{33}[S_2P(OPr)_2]_{17}$.

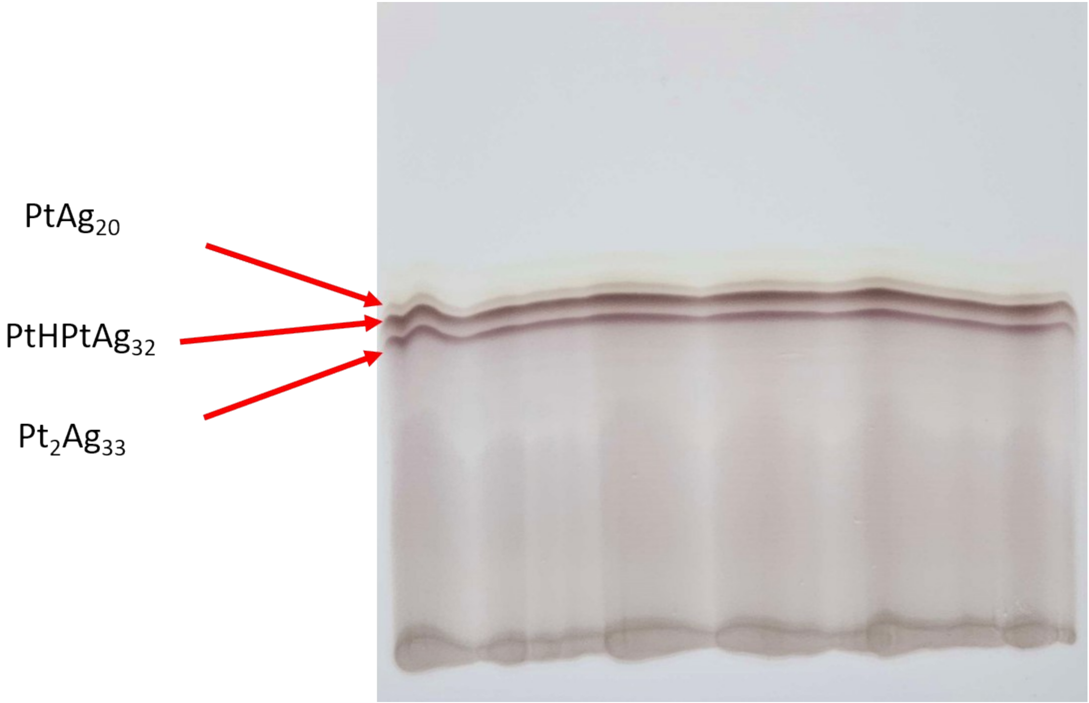

Figure S22. Photo of TLC under eluent combination: ether: hexane = 1:4.

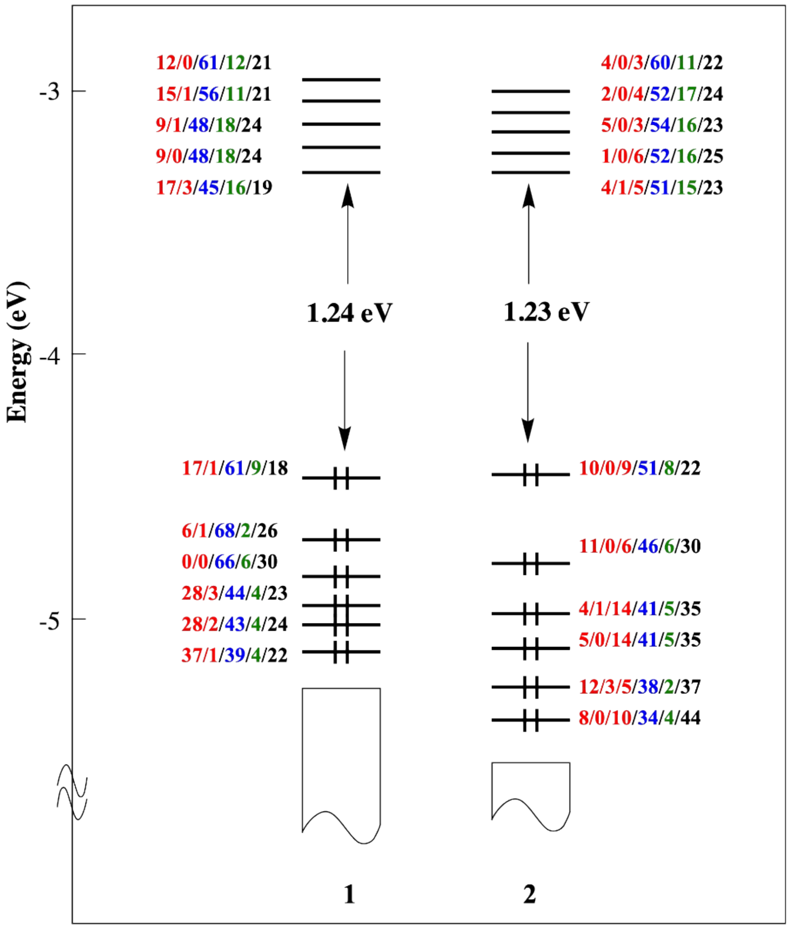

Figure S23. The Kohn-Sham frontier MO diagram of compounds **1** and **2** with orbital compositions as follows. **1**: **Rh_2/H_2/Ag_{23}(ico)/Ag_{10}(cap)/ligands**; **2**: **Pt/H/Pt/Ag_{23}(ico)/Ag_9(cap)/ligands**. The hydride of **2** lies in the left-side icosahedron.

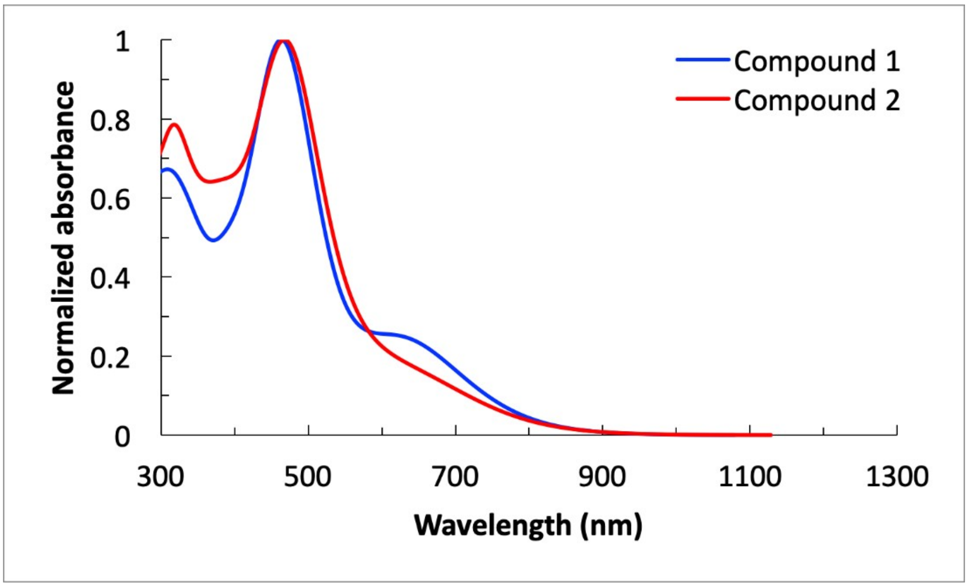

Figure S24. The TD-DFT-simulated UV-vis spectra of **1** and **2**.

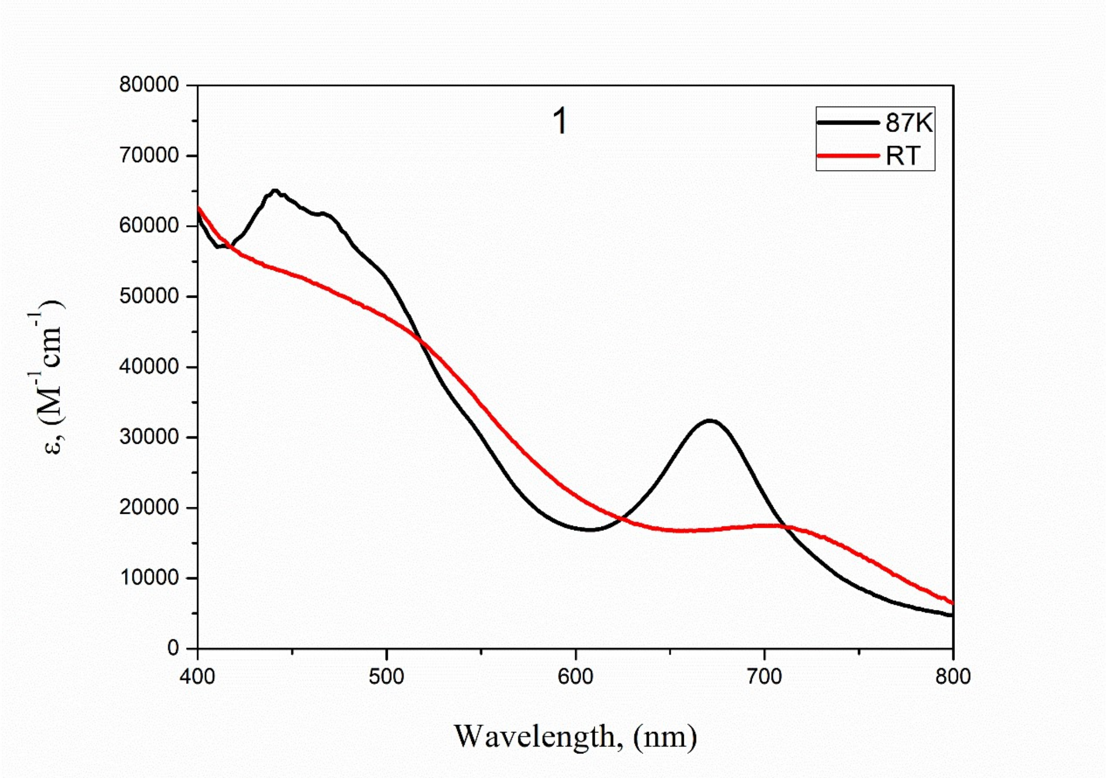

Figure S25. Low-temperature excitation spectra of Compound **1**.

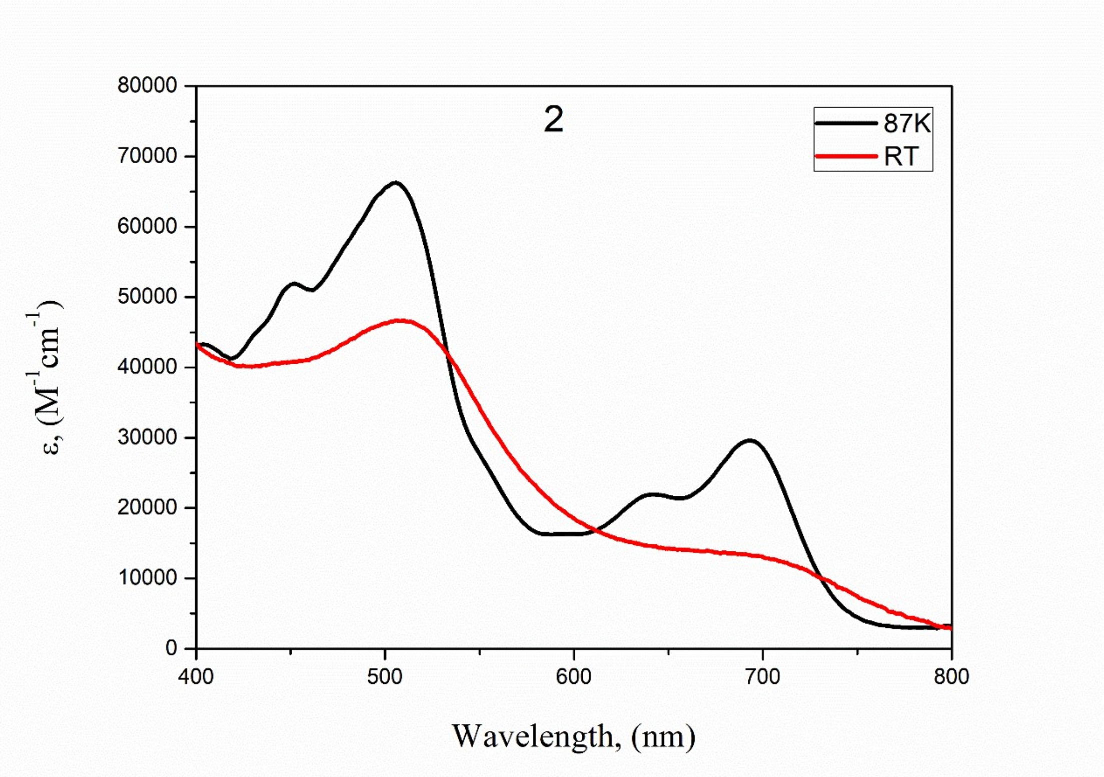

Figure S26. Low-temperature excitation spectra of Compound **2**.

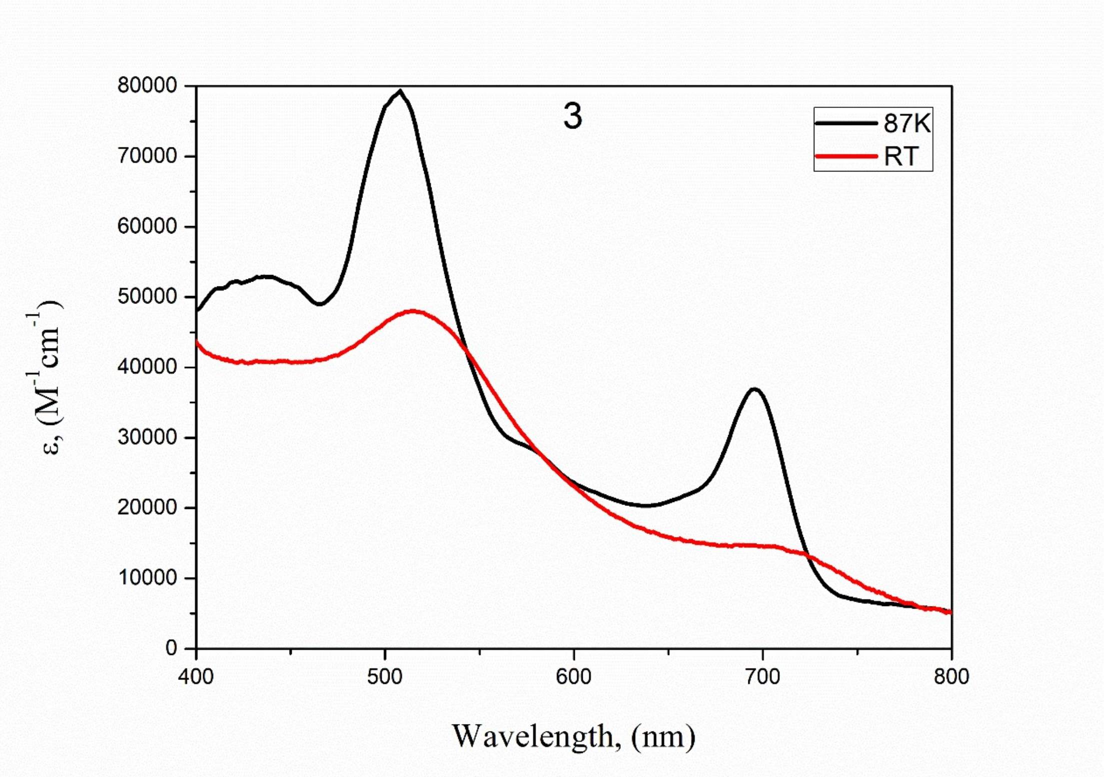

Figure S27. Low-temperature excitation spectra of Compound **3**.

Table S1. Selected X-ray crystallographic data of **1**,**2** and **3**.

Compound	**1**	**2**	**3**
CCDC no.	2342395	2342396	2342397
Chemical formula	$C_{204}H_{480}Ag_{66}O_{68}P_{34}Rh_4S_{68}$	$C_{102}H_{239}Ag_{32}O_{34}P_{17}Pt_2S_{34}$	$C_{102}H_{238}Ag_{33}O_{34}P_{17}Pt_2S_{34}$
Formula weight	14785.97	7468.47	7575.33
Wavelength, Å	0.71073	0.71073	0.71073
Crystal System	Triclinic	Triclinic	Monoclinic
Space group	$P\bar{1}$	$P\bar{1}$	$C2/c$
a, Å	16.193(2)	19.4846(11)	44.382(7)
b, Å	34.224(5)	20.7482(12)	17.680(3)
c, Å	38.997(5)	30.0519(17)	28.939(4)
α, deg.	72.855(4)	98.0114(14)	90
β, deg.	84.982(3)	102.1599(14)	111.569(5)
γ, deg.	83.671(2)	116.0390(13)	90
V, $Å^3$	20491(5)	10292.5(10)	21118(6)
Z	2	2	4
Temperature, K	100(2)	100(2)	100(2)
ρ_{calcd}, g/cm^3	2.396	2.410	2.383
μ, mm^{-1}	3.759	4.846	4.814
θ_{max}, deg.	25.000	25.000	25.000
Completeness, %	99.7	98.8	100
Reflection collected / unique	233268 / 71948 [R(int) = 0.0907]	67817 / 35841 [R(int) = 0.0445]	91475 / 18602 [R(int) = 0.0280]
Restraints / parameters	1470 / 4132	861 / 2070	359 / 1063
^{a} $R1$, ^{b} $wR2$ [I > 2σ(I)]	0.0666, 0.1254	0.0555, 0.1333	0.0364, 0.0733
^{a} $R1$, ^{b} $wR2$ (all data)	0.1174, 0.1519	0.0802, 0.1511	0.0448, 0.0811
GOF	1.103	1.032	1.113
Largest diff. peak and hole, e/Å^3	2.570 and -1.806	3.721 and -1.917	2.805 and -1.366

^{a} $R1 = \Sigma\,|\,|F_o| - |Fc|\,|\,/\Sigma|F_o|$. ^{b} $wR2 = \{\Sigma[w(F_o^2 - F_c^2)^2]/\Sigma[w(F_o^2)^2]\}^{1/2}$.

Table S2. Cartesian coordinates of the DFT-optimised structures of **1**, **2**, and **3**.

1

Ag	-0.010295	0.015327	-0.039281
Rh	-2.842274	0.027720	0.052654
Ag	-5.689982	0.194844	0.284859
Ag	-4.010817	2.572892	0.575700
Ag	-4.013423	0.465799	2.673238
Ag	-4.078994	-2.176638	1.257358
Ag	-4.417730	-1.575933	-1.718860
Ag	-4.154450	1.311926	-2.123495
Ag	-1.327861	-2.688926	-0.047693
Ag	-1.538361	-1.035542	2.335123
Ag	-1.482807	1.826784	1.821179
Ag	-1.553212	2.263943	-1.164577
Ag	-1.668868	-0.335833	-2.568506
Rh	2.836773	0.015214	0.041589
Ag	5.687163	-0.194789	0.280958
Ag	1.533660	-2.277573	-1.062943
Ag	1.471184	-1.694010	1.889255
Ag	1.539992	1.176119	2.287547
Ag	1.310114	2.733768	-0.154717
Ag	1.641169	0.284879	-2.550908
Ag	4.097565	2.248884	1.134677
Ag	4.448383	1.554531	-1.736236
Ag	4.119698	-1.345919	-2.094327
Ag	3.988100	-2.527912	0.654214
Ag	3.999159	-0.346455	2.674585
Ag	-6.776625	-1.372123	2.610093
Ag	-6.819600	-2.620878	-0.338460
Ag	-6.935332	0.081712	-2.428473
Ag	-3.848695	4.363803	-1.808892
Ag	-1.762101	4.667631	0.741574
Ag	1.768342	-4.605633	0.909236
Ag	6.918986	-0.160045	-2.418878
Ag	6.911692	2.590875	-0.424118
Ag	3.849083	-4.388103	-1.691910
Ag	6.775563	1.396327	2.570021
S	-8.016643	0.720349	1.583220

S	-8.934328	-1.242409	-1.255872
S	-7.822888	-3.700547	1.832873
S	-4.435624	-4.682471	1.771329
S	-5.550128	-1.757717	4.828476
S	-5.060799	1.711929	4.658157
S	-7.577560	2.597297	-2.513102
S	-4.435130	3.138013	-4.036115
S	-6.012502	-1.216385	-4.496272
S	-5.588091	-3.914279	-2.305926
S	-5.748141	4.573807	0.034379
S	-3.463813	5.073691	2.648012
S	0.059708	2.693058	3.840583
S	0.812964	4.972918	1.226143
S	-2.035372	6.204420	-1.437035
S	0.212775	3.676692	-2.548645
S	-0.053021	-2.487255	3.942995
S	-0.795622	-4.860290	1.409374
S	-1.731950	-1.155900	-4.958388
S	1.201083	0.784660	-4.965524
S	-0.220063	-3.736408	-2.418007
S	2.050508	-6.211914	-1.221825
S	5.758275	-4.522449	0.147466
S	3.503807	-4.962577	2.791413
S	4.371835	-3.225749	-3.955820
S	7.540413	-2.691116	-2.505355
S	7.998045	-0.732512	1.601843
S	8.973189	1.137728	-1.280864
S	4.491235	4.745949	1.640012
S	7.862652	3.692761	1.761767
S	5.664903	3.834092	-2.416129
S	6.083740	1.088000	-4.552324
S	5.072147	-1.653225	4.628099
S	5.541838	1.804893	4.779035
P	-9.403739	-0.054971	0.322011
P	-6.273273	-4.805956	2.573112
P	-5.658807	0.079608	5.673726
P	-6.481129	3.209924	-4.069515
P	-6.421878	-3.114800	-3.987062

P	-4.984341	5.744304	1.518950
P	0.900375	4.421947	3.188979
P	-0.334917	5.615205	-2.389692
P	-0.878755	-4.249309	3.347550
P	0.182570	-0.868387	-5.514988
P	0.351184	-5.664279	-2.200614
P	5.003712	-5.668552	1.654108
P	6.416299	-3.295021	-4.043439
P	9.410032	-0.004942	0.342071
P	6.316763	4.826728	2.473868
P	6.518711	2.986368	-4.062258
P	5.652595	-0.025144	5.644158
H	-10.383803	-0.749696	1.081145
H	-10.160504	1.033567	-0.178750
H	-6.677101	-6.170513	2.526079
H	-6.234014	-4.582981	3.975336
H	-6.979209	0.281736	6.167021
H	-4.954976	-0.040901	6.904635
H	-6.861695	4.542459	-4.376203
H	-6.853893	2.540885	-5.265172
H	-7.823749	-3.349304	-3.948282
H	-6.043006	-3.955683	-5.067743
H	-4.684898	7.023132	0.980501
H	-6.067117	6.062454	2.382309
H	2.266414	4.444536	3.576285
H	0.334508	5.473249	3.955772
H	-0.379048	6.146734	-3.702503
H	0.723294	6.367557	-1.816547
H	-2.245924	-4.291745	3.726787
H	-0.310046	-5.289731	4.128759
H	0.947785	-2.019811	-5.186041
H	0.225110	-0.922299	-6.933610
H	0.403143	-6.239425	-3.498374
H	-0.706426	-6.405702	-1.612704
H	4.695277	-6.959519	1.139790
H	6.092307	-5.979561	2.509658
H	6.748193	-2.610550	-5.243667
H	6.782642	-4.625082	-4.379765

H	10.165180	-1.118946	-0.107783
H	10.380209	0.699442	1.102225
H	6.750529	6.177062	2.428719
H	6.184929	3.822611	-5.159511
H	7.922555	3.195107	-3.984529
H	4.938901	0.070347	6.871700
H	6.967853	-0.242400	6.142447
H	6.253926	4.603042	3.879042
H	-2.483475	-1.292254	-0.890630
H	2.480709	1.311215	-0.944645

2

Pt	-2.998563	0.120179	0.036290
H	-3.056190	-0.891558	-1.319538
Pt	2.654767	-0.145138	0.181625
Ag	-5.868154	0.342218	0.272754
Ag	-4.200564	-0.343116	2.608030
Ag	-4.305601	-2.330997	0.338381
Ag	-4.745649	-0.470670	-2.309168
Ag	-4.157516	2.303904	-1.469858
Ag	-4.151726	2.398742	1.479268
Ag	-1.530715	-2.295554	-1.042008
Ag	-1.685135	-1.789839	1.936751
Ag	-1.683766	1.103599	2.401019
Ag	-1.537624	2.624783	-0.053769
Ag	-1.734154	0.746429	-2.411001
Ag	-0.166345	0.008244	0.081520
Ag	1.354354	-1.572962	-1.914894
Ag	1.243959	-2.505017	0.952927
Ag	1.243611	-0.102626	2.721899
Ag	1.439575	2.322645	0.899378
Ag	1.460335	1.460107	-1.896296
Ag	3.825305	-2.645710	-0.479134
Ag	3.766469	-1.657993	2.337700
Ag	3.838196	1.324246	2.292610
Ag	4.049214	2.164695	-0.599423
Ag	3.936678	-0.223377	-2.327978
Ag	5.501132	-0.330989	0.328303

Ag	-6.649062	-2.190254	2.283355
Ag	-7.205829	-2.023675	-1.055941
Ag	-3.426105	5.022777	-0.192412
Ag	-1.732605	3.806968	-2.814814
Ag	1.559639	-4.579777	-1.265785
Ag	3.710000	-3.129462	-3.426704
Ag	6.639584	1.090241	-2.005627
Ag	6.517200	2.520924	0.988457
Ag	6.578242	0.123535	3.120250
S	-7.951213	-0.061656	2.868207
S	-8.392653	0.287556	-0.599651
S	-8.026051	-3.821835	0.695509
S	-4.795697	-4.761837	-0.376472
S	-4.738606	-3.570825	3.394084
S	-4.685094	-0.546563	5.141203
S	-6.288861	3.456876	2.558326
S	-3.314734	5.240454	2.353826
S	-5.641199	4.628269	-1.500385
S	-3.837178	3.621227	-4.311144
S	-6.256520	-2.816258	-3.307456
S	-5.957774	0.447261	-4.426647
S	-1.051964	-4.888561	-1.039799
S	-0.336658	-4.108169	2.334561
S	-0.239700	-1.426546	4.530218
S	-0.416565	2.034584	4.416770
S	0.034750	4.476477	1.191404
S	-1.392766	6.117808	-1.548759
S	0.729613	3.203162	-3.672136
S	-1.525633	0.862953	-4.982825
S	-0.255878	-2.256652	-3.853827
S	1.982128	-5.028292	-3.874877
S	3.306864	-5.764069	0.238890
S	5.601885	-4.135885	-1.855704
S	4.931582	-3.616620	3.512105
S	5.204076	-0.547645	5.173793
S	4.073619	3.397191	3.784453
S	7.492445	2.596946	3.422959
S	5.814677	3.130821	-3.406314

S	5.217547	4.518302	-0.226927
S	4.180247	-1.001648	-4.850472
S	7.314808	-1.115382	-3.246694
S	7.847495	-1.275720	1.295274
S	8.677733	1.758995	-0.410019
P	-9.169888	0.042430	1.272798
P	-6.554301	-5.215596	0.485466
P	-4.417641	-2.538304	5.116024
P	-5.276436	5.154907	2.890798
P	-5.111568	4.918333	-3.444959
P	-6.828842	-1.351865	-4.601895
P	-1.199011	-5.305691	0.948129
P	-0.701375	0.321349	5.449657
P	0.240665	5.687626	-0.420694
P	0.174241	1.894617	-5.151360
P	0.274416	-4.098927	-4.486590
P	4.803385	-5.856338	-1.103363
P	5.355081	-2.569775	5.171829
P	5.914483	3.244388	4.554619
P	6.112247	4.588342	-2.063083
P	6.229581	-0.941151	-4.913982
P	9.192497	0.012157	0.488324
H	-10.068879	-1.058199	1.235347
H	-10.071906	1.119570	1.474494
H	-7.108756	-6.309637	-0.229505
H	-6.359307	-5.795592	1.767423
H	-5.193955	-3.113609	6.159912
H	-3.106671	-2.882813	5.542866
H	-5.317008	5.502105	4.269501
H	-5.971848	6.266976	2.338529
H	-4.681713	6.263402	-3.611214
H	-6.312181	4.929216	-4.201995
H	-8.246524	-1.231083	-4.592778
H	-6.638714	-1.876187	-5.910802
H	-2.582530	-5.443767	1.238389
H	-0.699480	-6.620094	1.133002
H	-2.037712	0.239114	5.930746
H	0.043045	0.408456	6.656943

H	0.779838	6.917574	0.035856
H	1.277811	5.216074	-1.268019
H	0.153817	2.640364	-6.359848
H	1.296993	1.051700	-5.371630
H	4.463061	-6.712196	-2.186484
H	5.875240	-6.580188	-0.518479
H	4.549708	-3.009030	6.258005
H	6.640636	-2.955367	5.644100
H	6.287912	4.513349	5.070953
H	5.917680	2.455888	5.736494
H	5.719949	5.821845	-2.647401
H	7.498765	4.823802	-1.857402
H	6.650697	-1.932743	-5.839228
H	6.536835	0.247475	-5.627972
H	9.961434	-0.736015	-0.439925
H	10.167896	0.323730	1.471171
H	-0.784582	-5.014537	-4.248634
H	0.295722	-4.070174	-5.905799

3

Ag	0.169517	5.656046	0.245258
Ag	-0.324210	3.970428	-2.292903
Ag	-2.380053	4.060431	-0.158337
Ag	1.909938	4.080698	2.051863
Ag	2.394318	4.021548	-0.883805
Ag	1.173175	1.469155	-2.020143
Ag	-1.896685	1.493158	-1.513627
Ag	-2.133470	1.503997	1.479205
Ag	-1.046084	4.035063	2.544713
Ag	2.597457	1.507509	0.651702
Ag	0.000000	0.000000	0.241603
Ag	0.556102	1.524399	2.772101
Ag	-1.702387	6.619882	-1.849623
Ag	2.386053	3.762268	-3.837060
Ag	4.234801	1.767572	-1.878404
Ag	-2.581699	6.569406	1.371091
Ag	0.139561	6.859198	3.028257
Pt	0.089336	2.816711	0.250543

Ag	-0.169517	-5.656046	0.245258
Ag	0.324210	-3.970428	-2.292903
Ag	2.380053	-4.060431	-0.158337
Ag	-1.909938	-4.080698	2.051863
Ag	-2.394318	-4.021548	-0.883805
Ag	-1.173175	-1.469155	-2.020143
Ag	1.896685	-1.493158	-1.513627
Ag	2.133470	-1.503997	1.479205
Ag	1.046084	-4.035063	2.544713
Ag	-2.597457	-1.507509	0.651702
Ag	-0.556102	-1.524399	2.772101
Ag	1.702387	-6.619882	-1.849623
Ag	-2.386053	-3.762268	-3.837060
Ag	-4.234801	-1.767572	-1.878404
Ag	2.581699	-6.569406	1.371091
Ag	-0.139561	-6.859198	3.028257
Pt	-0.089336	-2.816711	0.250543
S	-2.827864	4.237875	4.386399
S	-2.299239	7.666752	3.733207
S	-4.699750	5.136107	0.548053
S	-3.906537	5.651691	-2.849127
S	-2.218316	8.689905	-0.230817
S	1.114108	8.091274	0.924461
S	0.316156	7.348893	-3.344548
S	0.056703	4.204250	-4.915454
S	1.188512	5.651152	5.023014
S	3.952872	5.372007	2.883523
S	5.668668	3.577010	-0.691011
S	3.616820	5.751345	-2.537455
S	-1.663167	0.209046	-4.019166
S	-4.291371	-2.112851	-4.552215
S	-4.604351	0.830875	-1.608892
S	-4.320571	0.132027	1.851130
S	1.704913	0.380028	4.756679
S	-1.704913	-0.380028	4.756679
S	2.827864	-4.237875	4.386399
S	2.299239	-7.666752	3.733207
S	4.699750	-5.136107	0.548053

S	3.906537	-5.651691	-2.849127
S	2.218316	-8.689905	-0.230817
S	-1.114108	-8.091274	0.924461
S	-0.316156	-7.348893	-3.344548
S	-0.056703	-4.204250	-4.915454
S	-1.188512	-5.651152	5.023014
S	-3.952872	-5.372007	2.883523
S	-5.668668	-3.577010	-0.691011
S	-3.616820	-5.751345	-2.537455
S	1.663167	-0.209046	-4.019166
S	4.291371	2.112851	-4.552215
S	4.604351	-0.830875	-1.608892
S	4.320571	-0.132027	1.851130
P	-2.637438	6.122188	5.033202
P	-5.123183	5.942550	-1.284646
P	-0.367209	9.331252	0.301863
P	0.000000	6.249432	-4.984635
P	3.179103	5.816226	4.682719
P	5.464735	5.015140	-2.081740
P	-3.384767	-0.334196	-4.931299
P	-5.337032	0.924079	0.277550
P	0.000000	0.000000	5.768749
P	2.637438	-6.122188	5.033202
P	5.123183	-5.942550	-1.284646
P	0.367209	-9.331252	0.301863
P	0.000000	-6.249432	-4.984635
P	-3.179103	-5.816226	4.682719
P	-5.464735	-5.015140	-2.081740
P	3.384767	0.334196	-4.931299
P	5.337032	-0.924079	0.277550
H	-3.806865	6.465112	5.762322
H	3.806865	-6.465112	5.762322
H	-1.644485	6.224068	6.044416
H	1.644485	-6.224068	6.044416
H	-5.384287	7.325096	-1.084217
H	5.384287	-7.325096	-1.084217
H	-6.419526	5.476502	-1.629496
H	6.419526	-5.476502	-1.629496

H	-0.544212	10.348729	1.275490
H	0.544212	-10.348729	1.275490
H	0.157889	10.074523	-0.786467
H	-0.157889	-10.074523	-0.786467
H	-1.244942	6.559547	-5.594382
H	1.244942	-6.559547	-5.594382
H	0.907200	6.673751	-5.991560
H	-0.907200	-6.673751	-5.991560
H	3.624053	7.114826	5.057191
H	-3.624053	-7.114826	5.057191
H	3.799872	5.038252	5.697797
H	-3.799872	-5.038252	5.697797
H	6.256730	6.124766	-1.685707
H	-6.256730	-6.124766	-1.685707
H	6.112916	4.644671	-3.292032
H	-6.112916	-4.644671	-3.292032
H	-4.366549	0.675975	-4.752318
H	4.366549	-0.675975	-4.752318
H	-3.157907	-0.251474	-6.330418
H	3.157907	0.251474	-6.330418
H	-5.612997	2.287696	0.564780
H	5.612997	-2.287696	0.564780
H	-6.630524	0.341911	0.277520
H	6.630524	-0.341911	0.277520
H	-0.270444	1.058362	6.678270
H	0.270444	-1.058362	6.678270
